# Characterization of non-specific lipid transfer protein (nsLtp) gene families in the *Brassica napus* pangenome reveals abundance variation

**DOI:** 10.1186/s12870-021-03408-5

**Published:** 2022-01-07

**Authors:** Yu Liang, Yang Huang, Kang Chen, Xiangdong Kong, Maoteng Li

**Affiliations:** 1 Key Laboratory of Ecology of Rare and Endangered Species and Environmental Protection, Guangxi Key Laboratory of Landscape Resources Conservation and Sustainable Utilization in Lijiang River Basin, College of Life Science, Guangxi Normal University, Guilin, China; 2 School of Mechanical and Electrical Engineering, Guilin University of Electronic Technology, Guilin, China; 3 Department of Biotechnology, College of Life Science and Technology, Huazhong University of Science and Technology, Wuhan, China

**Keywords:** nsLTP, *Brassica napus*, Pangenome, cis-elements, Gene expression, QTL

## Abstract

**Background:**

*Brassica napus* is an important agricultural species, improving stress resistance was one of the main breeding goals at present. Non-specific lipid transfer proteins (nsLTPs) are small, basic proteins which are involved in some biotic or abiotic stress responses. *B. napus* is susceptible to a variety of fungal diseases, so identify the *BnLTPs* and their expression in disease responses is very important. The common reference genome of *B. napus* does not contain all *B. napus* genes because of gene presence/absence variations between individuals. Therefore, it was necessary to search for candidate *BnLTP* genes in the *B. napus* pangenome.

**Results:**

In the present study, the *BnLTP* genes were identified throughout the pangenome, and different *BnLTP* genes were presented among varieties. Totally, 246 *BnLTP* genes were identified and could be divided into five types (1, 2, C, D, and G). The classification, phylogenetic reconstruction, chromosome distribution, functional annotation, and gene expression were analyzed. We also identified potential cis-elements that respond to biotic and abiotic stresses in the 2 kb upstream regions of all *BnLTP* genes. RNA sequencing analysis showed that the *BnLTP* genes were involved in the response to *Sclerotinia sclerotiorum* infection. We identified 32 *BnLTPs* linked to blackleg resistance quantitative trait locus (QTL).

**Conclusion:**

The identification and analysis of *LTP* genes in the *B. napus* pangenome could help to elucidate the function of *BnLTP* family members and provide new information for future molecular breeding in *B. napus*.

**Supplementary Information:**

The online version contains supplementary material available at 10.1186/s12870-021-03408-5.

## Background

Non-specific lipid transfer proteins (nsLTPs) are small, basic proteins that are involved in physiological and biochemical reactions in plant growth, including lipid transport between cell membrane systems, inhibiting the growth of pathogens and signalling. *nsLTPs* are low-molecular-weight cysteine-rich proteins and are widely distributed in the plant kingdom [1]. Their sequences are characterized by an 8-cysteine motif (ECM): C-Xn-C-Xn-CC-Xn-CXC-Xn-C-Xn-C [2]. The N-terminal of mature *nsLTPs* contains a signal peptide typically composed of 21–29 amino acid residues. In addition, some lipid transfer proteins (LTPs) also carried the motifs that add glycosylphosphatidylinositol (GPI)-anchors, which attach proteins to the outside of the plasma membrane [3]. The main feature of the spatial structure of plant *nsLTPs* is that H1–H4 are connected by four pairs of disulphide bonds that form a stable structure with a hydrophobic hole [4]. The hydrophobic parts of some lipids can interact with this hole. To date, many kinds of LTP genes have been isolated in many plants. Boutrot et al. had divided the LTPs into nine types according to sequence similarity [2]. Edstam et al. further classified the plant LTPs into four categories (1, 2, D, G) and several subclasses (C, E, X, etc.) according to the intron position, sequence identity, the distance between cysteine residues in the ECM, and the post-translational modifications [3].

The *nsLTP* family is ubiquitous and has been studied in many plants, such as in *Arabidopsis thaliana*, rice, *Triticum kiharae* and *Brassica rapa* [2, 5, 6]. *nsLTPs* are involved in a variety of physiological functions, such as the ability to enhance cell wall extension in tobacco and wheat [7–10]. In addition, Zhao et al. found that overexpression of the *OsLTPL* 159 ^IL 112^ could enhance the cold tolerance of rice [11]. Transgenic plants overexpressing the *StnsLTP1* gene acquire improved tolerance to multiple abiotic stresses through enhanced activation of antioxidative defence mechanisms [12]. Dhar et al. found an *nsLTP* gene was down-regulated in response to multiple bacterial pathogens, further research revealed that this *nsLTP* was required for defence against bacterial and fungal pathogens as well as for normal seedling growth under salinity stress, and it was named as disease-related non-specific lipid transfer protein 1 (DRN1, 13]. Although its molecular mechanism is not clear, the responses of LTP genes in these studies suggested that nsLTPs are played an important role in plant stress resistance.

*Brassica napus* is an important oil crop globally. Only a few studies have focused on single genes in nsLTP family, such as *BraLTP1*, a new nsLTP gene involved in wax production or deposition, it also has direct or indirect effects on cell division and flower development [13]. Another study found that a new nsLTP gene *BraLTP2* might be involved in trichome development and the accumulation of secondary metabolites [14]. However, there has been no genome-wide analysis of nsLTP family in *B. napus*. After the reference genome of rapeseed cultivar ‘Darmor-bzh’ was published in 2014, a higher-quality genome of the cultivar ‘ZS11’ was released in 2017 [15, 16]. However, owing to the existence of structural variations, especially the presence/absence variations (PAV), the genome of a single cultivar cannot completely cover the entire gene catalogue. Pangenomes have been constructed for many species to obtain more genetic data resources, such as soybean, maize, rice, tomato [17–21]. In 2019, Song et al. constructed a high-quality pangenome of *B. napus* by using eight *B. napus* varieties [22, 23]. In the present study, the nsLTP gene identification, characterization, and expression analysis were conducted by whole-pangenome, and a total of 246 nsLTP family genes were identified, and which could be classified into five types: 1, 2, C, D, G. We classified the nsLTPs belonging to the reference genome and non-reference regions, and present a more comprehensive understanding of nsLTP distribution in different varieties. The phylogeny, Ka/Ks, GO enrichment and expression of the nsLTP family were also analyzed. These results lay a foundation for further studies on the function of the LTP family in *B. napus*.

## Method

### Identification of nsLTP genes in the *B. napus* pangenome

The pangenome sequence was downloaded from the *B. napus* pangenome information resource (http://cbi.hzau.edu.cn/bnapus/) [22]. The coding sequence (CDS) and protein sequences of the pangenome were obtained by gffread v0.12.7 with the pangenome sequences and annotations [24]. NsLTP sequences of *A. thaliana* were downloaded from TAIR (http://www.arabidopsis.org). The homologous nsLTPs genes between *A. thaliana* and *B. napus* were identified by comparing the CDS sequences to the *A. thaliana* nsLTPs sequences with blastx v 2.2.26 using a threshold of 1E–5. The Tryp_alpha_amyl.hmm and LTP_2.hmm files in pfam [25] were downloaded, and hmmscan v3.3.1 was used to identify candidate LTP family genes with a threshold of 1E–5. After finding the candidate nsLTPs in two ways, the protein sequences were submitted to the conserved domain database (CDD) [26] and InterProScan (https://www.ebi.ac.uk/interpro/search/sequence/) to check for the presence of LTP domains.

An in-house perl script was then used to check for the presence of the ECM structure in the candidate LTP, and sequences with no ECM structure were removed. Then, we used SignalP v4.1 to predict the location of the signal peptides [27]. Sequences without signal peptides were also removed.

### NsLTP sequence analysis

Two prediction tools, PredGPI (http://gpcr2.biocomp.unibo.it/gpipe/pred.htm) and GPI-SOM (http://gpi.unibe.ch/), were used to determine the presence of C-terminal GPI-anchor sites. Sequences with GPI-anchor sites were classified as category G, and the others were classified according to Edstam et al. [3]. *A. thaliana* nsLTP sequences were downloaded from TAIR, and *B. napus* LTP sequences were used to construct a phylogenetic tree. The multiple sequence alignment tool MEGA7 was used to generate a phylogenetic tree by the neighbour-joining method. MEME v2.0.0 [28] was used to analyse the conserved motifs in the *B. napus* nsLTP genes, with the highest number of motifs set to 10. Gene structures were visualized using GSDS 2.0 [29].

### Gene duplication analysis, synonymous and non-synonymous substitution rates estimation

The duplication of genes can be divided into two types: tandem duplication and segmental duplication. The criterion for tandem duplication of LTP gene is that the physical distance of two genes on the chromosome should fall within 50 kb [30].

Segmental duplication refers to the case where the two genes are physically far away on the chromosome, but are still homologous. The criteria are as follows: (1) the region of overlap between homologous genes accounts for more than 80% of the total length of longer genes and (2) the sequence similarity is more than 80%. Synonymous and non-synonymous substitution rates of duplicated genes were calculated by TBtools v1.068 [31].

### Cis-element analysis of the promoter regions of the nsLTP genes

In this study, in-house perl script was used to extract the upstream 2 kb sequence of *BnLTP* genes. The binding site prediction tool (http://plantregmap.gao-lab.org/binding_site_prediction.php in plantregmap) was used to predict TF binding sites in the 2 kb upstream sequence of *BnLTPs*.

### Gene ontology (GO) annotation and enrichment analysis

All *BnLTP* sequences were compared with sequences in the uniprot database using blastp with a threshold of 1E-5. Using the Retrieve/ID mapping tool (https://www.uniprot.org/uploadlists/), uniprot IDs were converted to GO IDs to perform GO annotation for *BnLTPs*. AgriGO (http://bioinfo.cau.edu.cn/agriGO/analysis.php) was used for GO enrichment analysis. All genes of the *Brassica napus* pan-genome were used as the background gene set.

### RNA-seq data analysis

The RNA-seq data were downloaded from NCBI with the SRA accession number SRP053361(https://www.ncbi.nlm.nih.gov/sra/?term=SRP053361, 33]. Fastq-dump of SRA Toolkit v2.10.0 (http://www.ncbi.nlm.nih.gov/Traces/sra/sra.cgi?view=toolkit_doc&f=fastq-dump) was used to convert the downloaded SRA file to FASTQ Format file. Fastp v0.20.1 was used to remove adapters and low-quality sequences in the original data.

The RNA-seq clean data of each sample were aligned to the *Brassica napus* pan-genome using HiSAT2 v2.1.0. The FPKM (Fragments Per Kilobase of transcript per Million mapped reads) values of genes in all samples were calculated as the expression level. The DESeq2 v1.32.0 [32] was used for differential expression analysis, and unigenes with |log2 ratio| ≥ 1 and q < 0.05 as a cut-off for significant differential expression were selected. The transformed log2 (FPKM + 1) values were used to generate the heatmap using R (version 4.0.3) with the ComplexHeatmap package (version2.6.2, https://bioconductor.org/packages/release/bioc/html/ComplexHeatmap.html).

### Linking known QTL and BnLTP genes

Gene and QTL region overlap was determined using bedtools v2.16.2 intersect [33]. Known blackleg resistance-linked QTL were collected from the literature [34–38]. Waterfall plots were drawn using Variant Effect Predictor v99 [39], GenVisR v1.11.3 [40], vcftools v0.1.16 [41].

## Results

### Pangenome-wide identification and distribution of nsLTPs in *B. napus*

Song et al. sequenced and assembled high-quality genomes of eight varieties: ZS11, Gangan (GG), Zheyou7 (ZY), Shengli (SL), Tapidor (TA), Quinta (QU), Westar (WT), and No2127 (NO) [22], combined with the genome of ‘Darmor-bzh’ [15], and the pangenome of *B. napus* was constructed. Totally, 246 *BnLTP* sequences with an ECM domain were obtained, including 115 G typeGPI-anchored nsLTPs (detected by GPI-SOM and PredGPI) and 1, 2, C and D types of 29, 50, 4 and 48 without a GPI-anchored nsLTPs (the type-specific cysteine spacing patterns suggested by Edstam et al.) (Table 1).

**Table 1:** Characteristics of different types of *B. napus* non-specific lipid transfer proteins (nsLTPs)

Type	Number of members	GPI-anchor	Cysteine Spacing Pattern						
1	29	NO	C	X9	C13,14	CC	X19,20	CXC	X19,21–24
2	50	NO	C	X7	C13,14	CC	X8	CXC	X10,19,21,23,25
C	4	NO	C	X9	C16	CC	X9	CXC	X12
D	48	NO	C	X9,10,13	C12,14–19	CC	X9,12,13	CXC	X19,22,24,26
G	114	YES	C	X6,7,9,10,11,13	C11,13–18	CC	X8,9,12-14,19	CXC	X19,22–26,19

**Fig. 1:**
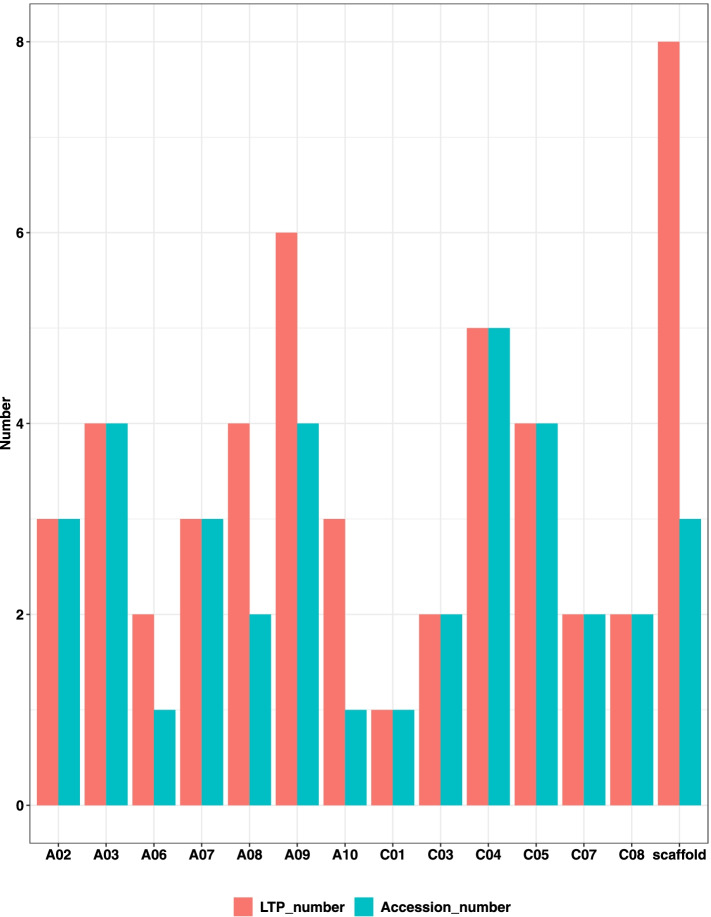
Distribution of *BnLTPs* in presence/absence variation regions of the chromosomes in *B. napus*

In the pangenome-wide search for nsLTP genes, *BnLTP* genes were identified on all chromosomes in the reference genome of ZS11 (Table S1), in which, 197 were located in the reference genome of ZS11 and 49 were located in non-reference regions. It was revealed that the *BnLTPs* were unevenly distributed on different chromosomes, C02 and C03 were with 18 *BnLTPs*, and only two LTPs were located on A04. According to PAV data, the LTP family genes identified in non-reference genomes were distributed across 13 chromosomes and A09 was with the most numbers (Fig. 1). The non-reference regions contained 1 (1), 2 (7), D (13) and G (23) types of *BnLTPs* with the number of 1, 7, 13 and 23, respectively. According to the PAV information, the *BnLTPs* were not evenly distributed in all varieties (Table 2), and the variety GG had the most of 13 *BnLTP* genes, otherwise, only 1 was observed in QU, it indicated that *BnLTP* genes may be lost in QU and duplicated in GG during domestication. The above results also suggested that when analysing the nsLTP gene family should not only focus on the reference genome.

**Table 2:** Count of BnLTP genes per variety and per BnLTP type

Type	D	GG	NO	QU	SL	TA	WE	ZY
1	0	1	1	0	0	1	1	2
2	2	2	2	0	1	0	0	0
C	0	0	0	0	0	0	0	0
D	1	4	2	0	4	1	0	1
G	1	6	4	1	2	1	2	5

### Phylogenetic analysis and BnLTP sequence features of the *B. napus*

**Fig. 2:**
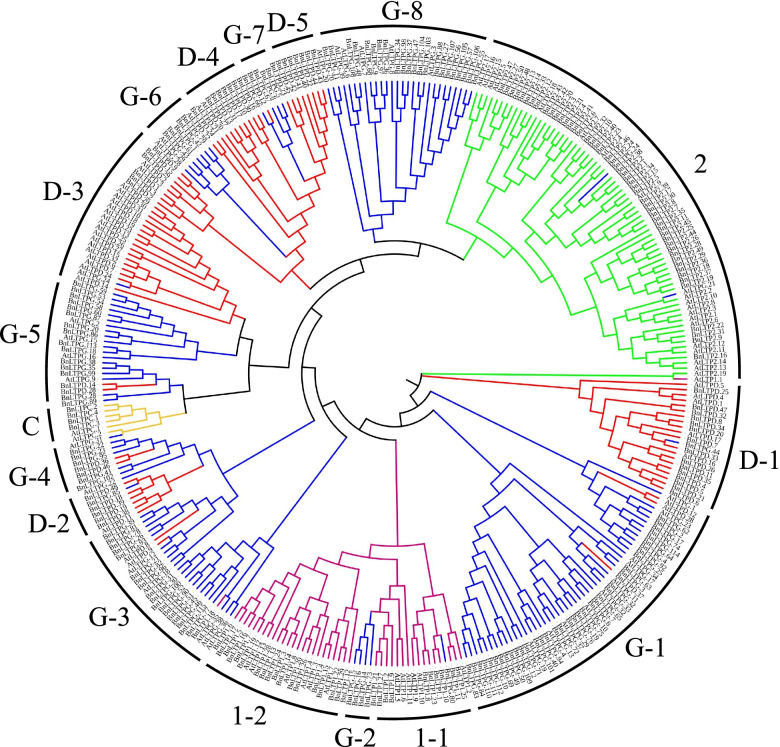
A phylogenetic tree of the putative nsLTPs from *Brassica napus* and *Arabidopsis thaliana*. The phylogenetic tree was constructed by *BnLTP* amino acid sequences. Different types were marked with different colours. Bootstrapping was carried out 10,000 times to get support values for each branch

The neighbour-joining method was used to construct a phylogenetic tree based on the nsLTP sequences of *B. napus* and *A. thaliana*. The branching of the tree is consistent in general with the classification into the main structural types and indicates that *nsLTP* genes in each clade were originated from a common ancestor (Fig. 2), further analysis revealed that the types1, D, and G have two, five and eight sub-branches, respectively. According to Fig. 2, the typeD and G *BnLTPs* are closely related, since many sub-branches of typeD and G are very close in the phylogenetic tree.

**Fig. 3:**
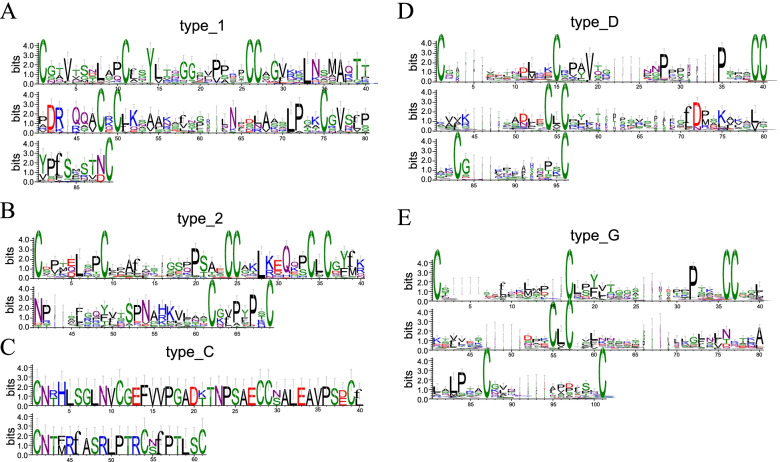
Sequence logo plots of an 8-cysteine motif (ECM) region sequences for each *Brassica napus* lipid transfer proteins type. Larger amino acid residues in the picture indicate higher sequence conservation level

ECM sequence logos were constructed for each *BnLTP* type to explore which amino acid residues were conserved between adjacent cysteine residues. Figure 3 shows that type1, 2, and C are more conservative than that of typeD. The CXC structure can affect cysteine pairing and influence protein folding. Among all the *BnLTP* sequences, there were 12 different amino acid residues in the CXC structure: A, E, F, G, I, K, L, M, Q, R, V, and Y. Hydrophobic residue L was the most common residue in type2, D, and G. In type1, the proportions of R, K, Q, and E residues were similar.

### Duplication patterns and synteny analysis of the BnLTP genes

**Fig. 4:**
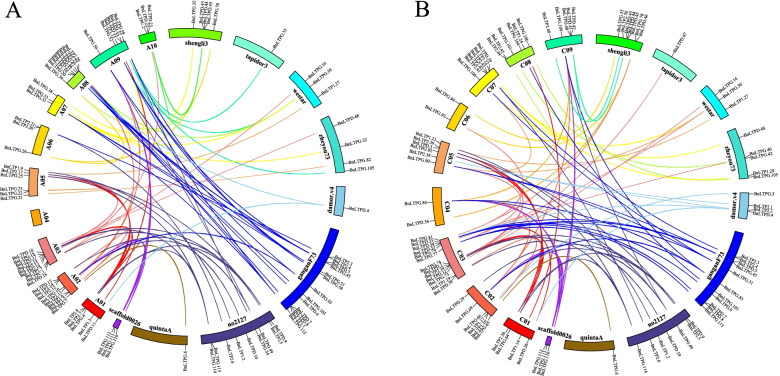
**A**, Distribution and syntenic relationship of the *BnLTP* genes in the *B. napus* A genome and pan-genome additional contigs. **B**, Distribution and syntenic relationship of the *BnLTP* genes in the *B. napus* C genome and pan-genome additional contigs

Gene duplication is considered to be a driving force in evolution and an important factor to increase genome complexity. *B. napus* is an allotetraploid formed by natural interspecific hybridization and chromosome doubling between *B. oleracea* (CoCo, 2*n* = 18) and *B. rapa* (ArAr, 2*n* = 20, 16]. Therefore, there are usually two or more duplicated gene sequences in the genome of *B. napus*. Segmental and tandem duplications are considered to be the main reason why the gene family is expanded. In the present study, 22 pairs of tandem duplications were identified (Table S2), including two pairs in GG, three in SL, and 17 in ZS11. Among them, the A10 chromosome of Shengli had the highest number of tandem duplication events. It was revealed that three pairs of tandemly duplicated *BnLTP* genes (*BnLTPD.43*, *BnLTPD.44*, *BnLTPD.45*) were all in the same duplicated gene blocks. Segmental duplications were identified by blastp with the requirement that there was > 80% overlap with the longer sequence, > 80% sequence similarity, and the repeated pairs are counted only once. A total of 339 segmental duplications *BnLTP* pairs (Table S3) were identified, including of 4 genes in D, 2 in TA, 1 in QU, 9 in NO, 3 in WE, 13 in GG, 7 in ZY and 7 in SL. Among them, the C03 chromosome of ZS11 had the highest number of segmental duplication events. We also analyzed the synteny of the *BnLTP* genes between the reference genome and non-reference regions (Fig. 4). Many variety-specific *BnLTP* genes were with a syntenic relationship with the *BnLTP* genes on the reference genome, such as 13 *BnLTP* in Gangan have syntenic relationship with the *BnLTP* genes on chromosomes A06, A07, A08 and A09 of the reference genome, and 11 Gangan-specific *BnLTPs* have a syntenic relationship with the *BnLTP* on the reference chromosomes of C04, C05, C07, C08 and C09 of *B. napus* C genome. In the varieties Quinta and Tapidor, only one specific gene maintained a syntenic relationship with *BnLTP* on the reference genome. This indicated that *BnLTPs* have different duplication patterns in different varieties.

### Cis-regulatory factors and GO analysis of the LTP gene

**Fig. 5:**
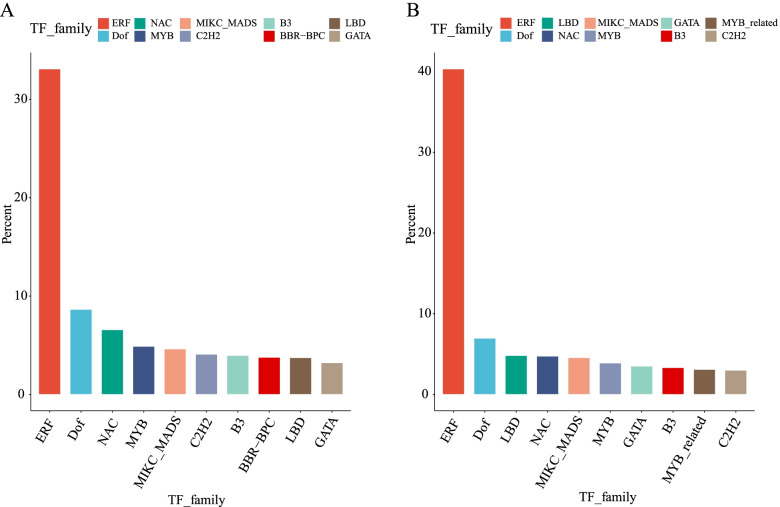
The top 10 cis-elements identified in 2 kb upstream regions of *BnLTP* genes. **A** Upstream regions of the genes in the reference genome. **B** Upstream regions of the genes in the pan-genome additional contigs.

Analysis of the 2 kb upstream regions (from the translation start site) of *BnLTP* genes could reveal the presence of various regulatory elements that are associated with the development and abiotic/biotic stress signalling. It was revealed that the ethylene-responsive factor (ERF) binding sites account for the largest proportion of sites in the upstream regions of the genes in the reference genome and non-reference region (Fig. 5), these sites are involved in hormonal signal transduction, response to biotic and abiotic stresses, regulation of metabolism, and developmental processes in various plant species. Weidong Wang et al. found that the upstream of LEA gene family in tea species is regulated by ERF, MYB and bZIP and bHLH transcription factor [42], which shows that the genes regulated by AP2/ERFs is a common phenomenon. Some other regulatory elements involved in the regulation of growth and development processes, such as the Dof (DNA binding with one finger) binding site, were also enriched in *BnLTPs*. The pathogen- and viral infection-responsive cis-regulatory elements included of the TF binding sites NAM, ATAF, and CUC (NAC) were also identified. Other regulatory elements, such as binding sites for C2H2 zinc finger transcription factor (involved in the occurrence of plant leaves and the regulation of floral organs) and lateral organ boundaries domain (LBD, involved in the development and formation of plant lateral organs), also existed.

**Fig. 6:**
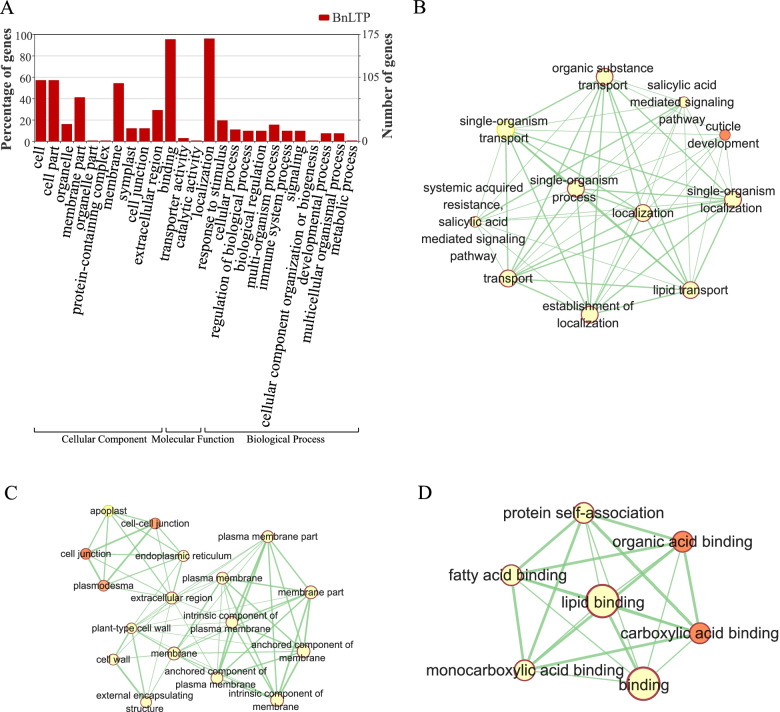
GO term annotation classification of *BnLTPs*(**A**) and the enriched Gene Ontology (GO) terms in molecular function (**B**), biological process (**C**) and cellular component (**D**) categories

To further understand the function of LTP in organisms, GO annotation and enrichment analysis of LTP genes were performed. Figure 6A shows that binding (GO:0005488) was the most abundant GO term that LTPs were identified as being involved in, which is consistent with the function of *BnLTP*. In the GO function enrichment analysis, significant enrichment was observed for a large number of GO terms related to binding and transport, such as lipid binding, fatty acid binding, monocarboxylic acid binding, lipid transport, and organic substance transport. Meanwhile, systemic acquired resistance and salicylic acid-mediated signalling pathways were also significantly enriched, which reveals that the nsLTP gene family was involved in biotic and abiotic stress responses. (Fig. 6B, C, D).

### The expression of BnLTP genes that response to Sclerotinia sclerotiorum

**Fig. 7:**
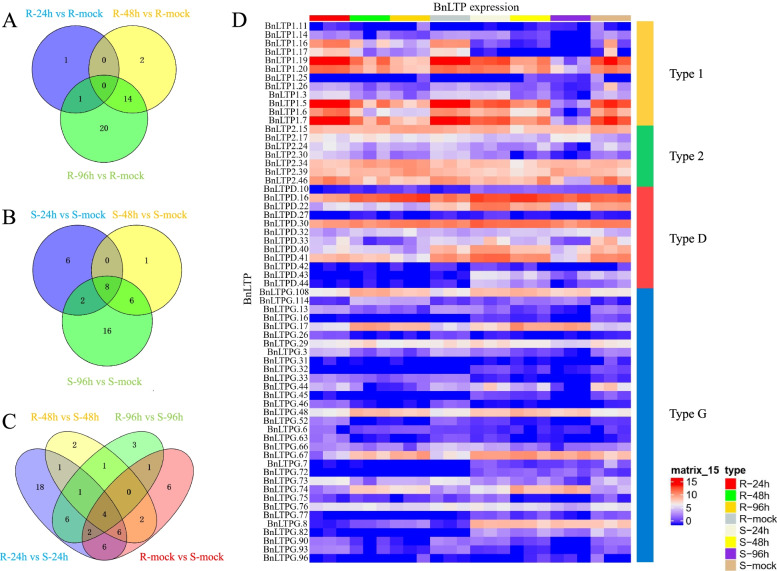
Transcriptome analysis of *BnLTP* responses to *Sclerotinia sclerotiorum.* **A**, **B**, **C** Venn Diagrams of overlapping and specific differentially expressed *BnLTP* genes for different comparisons. **D** Heatmap of the expression of the differentially expressed *BnLTP* genes

Sclerotinia stem rot, caused by *S. sclerotiorum*, is one of the most destructive diseases in rapeseed. Wu et al. studied the transcriptional response of rapeseed under the resistant line (R-line) and a susceptible line (S-line) [43]. Compared to the mock-inoculated samples, only two *BnLTPs* were differentially expressed in R-line in 24 h after infection, while in S-line, 16 *BnLTP* genes were differentially expressed (Fig. 7A) in 24 h after infection. After 48 and 96 h of infection, the R and S lines showed similar numbers of differential expression genes. However, according to Fig. 7A and B, the response of *BnLTPs* to inoculated *S. sclerotiorum* was different at different periods in the two lines. In the R-line, there were no common differentially expressed *BnLTP* genes between the three periods, and 20 *BnLTP* genes were only found in 96 h after infection. In the S-line, only eight *BnLTP* genes were found in all three periods, and 16 *BnLTP* genes were only in 96 h after infection. The two lines were compared at different infection stages, and there were 44, 17, and 18 differentially expressed *BnLTP* genes 24, 48, and 96 h after infection (Fig. 7C), respectively, indicating that the response of *BnLTP* genes in the two varieties was the most different in the early stage of infection.

Log^2^ (FPKM+ 1) values of the genes were used to plot the heatmap (Fig. 7D), it shows that some typeG *BnLTP* genes exhibited low expressions, but exhibited most differential expression. There were fewer type1 *BnLTP* genes than type2 *BnLTP* genes in the pangenome, but more differentially expressed type1 *BnLTP* genes than type2 *BnLTP* genes. Figure 7D shows the expression level of each differentially expressed *BnLTP* gene in the different samples. The expression of *BnLTPG.7* and *BnLTPG.72* genes was lower in the R-line than in the S-line.

### Linking BnLTP genes to known QTL for blackleg resistance

To further assess possible biological functions of *BnLTPs*, we compared the position of *BnLTPs* with known QTL for blackleg resistance [44]. Positions were predicted from known genomic regions of seven loci in the ZS11 assembly. The QTL name and references are shown in Table 3. These QTLs covered between 5.3 Mb and 30.5 Mb on three pseudomolecules (Rlm1, Rlm3, Rlm4, Rlm7 and Rlm9 were located on A07, as well as LepR1 and LepR2 was located on A02 and A10 respectively). Furthermore, the mapping of Rlm4 covered the greatest number of *BnLTPs* which 1689 consist of 4 core and 4 variable genes. Rlm7 and Rlm9 loci contained only one variable *BnLTP*. The Rlm1 was the narrowest QTL but contained 5 *BnLTPs* which number was in the medium of all loci.

**Table 3:** BnLTPs underlying reported QTL for blackleg in the ZS11 assembly

Locus	Resistance	Publication	Pseudomolecule	Start	End	Length	Core genes percentage
				(bp)	(bp)	(bp)	
*Rlm1*	blackleg	Delourme et al., 2004	A07	25,515,316	30,891,516	5,376,200	3 core, 2 variable
		Raman et al., 2012b					(60% core)
*Rlm3*	blackleg	Delourme et al., 2004	A07	11,368,284	29,211,062	17,842,778	1 core, 3 variable
		Leflon et al., 2007					(25% core)
*Rlm4*	blackleg	Raman et al., 2012a	A07	1,512,732	32,016,319	30,503,587	4 core, 4 variable
		Tang and Zhao, 2015					(50% core)
*Rlm7*	blackleg	Larkan et al., 2016	A07	15,452,927	23,156,986	7,704,059	0 core, 1 variable
							(0% core)
*Rlm9*	blackleg	Delourme et al., 2004	A07	17,146,407	22,717,328	5,570,921	0 core, 1 variable (0% core)
*LepR1*	blackleg	Larkan et al., 2016	A02	10,497,184	23,959,094	13,461,910	1 core, 6 variable (14.29% core)
*LepR2*	blackleg	Larkan et al., 2016	A10	253,009	20,875,594	20,622,585	3 core, 3 variable
							(50% core)

**Fig. 8:**
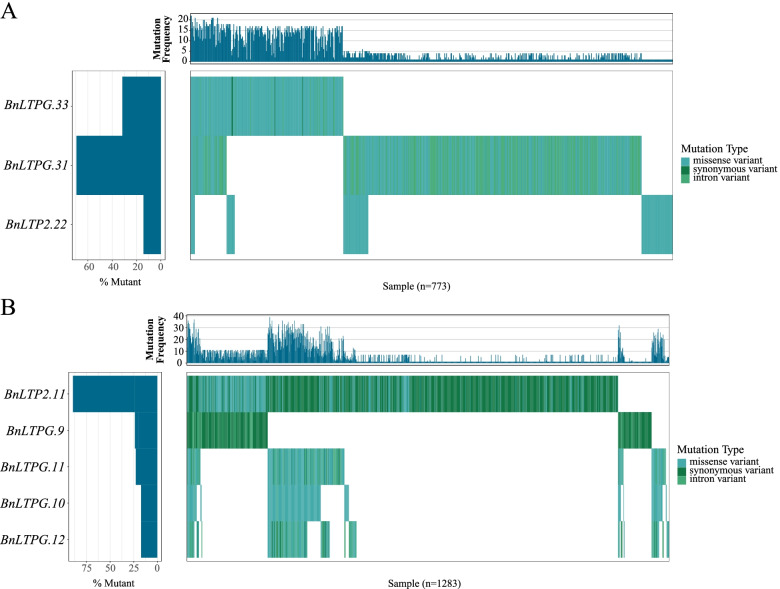
Waterfall plot of the blackleg-linked QTL. **A** Waterfall plot of Rlm3 and Rlm4 loci. **B** Waterfall plot of LepR1 locus. The variations were identified by combined 1689 rape resequencing data produced by several studies. The sample sizes in the figure means that mutations were identified in the *BnLTP* genes located in the QTL regions in these samples

Song et al. combined rape resequencing data produced by several studies, then a large number of variations were identified [23]. Based on the mutation information, two waterfall plots of the blackleg-linked QTL (LepR1 locus, Rlm3 locus and Rlm4 locus) were produced to show the mutational load of *BnLTPs* located within the QTL candidate regions. We filtered *BnLTPs* without SNP and individuals without mutation in *BnLTPs* and finally showed the mutation load of 773 individuals in Rlm3 and Rlm4 loci as well as 1283 individuals in LepR1. Moreover, the loci of Rlm3 and Rlm4 covered the same *BnLTPs* thus their waterfall plots are identical (Fig. 8). As shown in the waterfall plot of Rlm3 and Rlm4 loci, the maximum and minimum mutation percentages were related to *BnLTPG3.1* and *BnLTP2.22*, respectively (Fig. 8A). *BnLTP2.11* exhibited the highest mutation in all *BnLTPs* located on LepR1 locus, and mainly observed synonymous variant which has very few low impacts.

## Discussion

In this study, 246 *BnLTP* genes were identified, including of 29 type1, 50 type2, four typeC, 48 typeD, and 114 typeG in the entire pangenome of rapeseed. In addition to the 197 *BnLTPs* found in the reference genome, another 49 *BnLTPs* in the non-reference region of the non-reference sequences also existed. In the study of Hurgobin et al. [45], 53 synthetic and nonsynthetic accessions were used to generate the first *B. napus* pangenome. 13,631 new genes were predicted in the pangenome. We compared the 13,631 genes with the pan-genome published by Song et al. [22] using the software BLASTN with the parameters’- e 1emur10’ and’- F F ‘and a sequence identity of > 98%. Finally a new *nsLTP* gene was identified uniquely in the 13,631 genes. It indicates that more members of gene family may be found in the genomic data resources of more varieties, and more *nsLTP* genes can be discovered on the basis of this study in the future.

In different cultivated species, the distribution of *BnLTPs* in the non-reference region was not balanced, which indicated that the different varieties underwent different forms and levels of selection during domestication. In the reference genome, the identified *BnLTPs* were unevenly distributed across the genome. The uneven distribution of *BnLTPs* might be due to recent *BnLTP gene* tandem duplications, segmental duplications and dosage compensation, which is critical for development and phenotypic characteristics [44]. The results show that the order and proximity of genes of the gene family are important to the functional nature of these genes [44].

In *B. oleracea*, Bayer et al. revealed that the single nucleotide polymorphisms (SNPs) and presence-absence variation (PAV) of *B. oleracea* have experienced different selection pressures for different resistance gene analog (RGA) classes [46]. Dolatabadian et al. identified 1749 RGAs in the *B. napus* pangenome, 996 of which are core, 753 are variable, and 368 are not present in the reference genome [45]. The present study and the above mentioned two pangenome studies showed that it is necessary to identify candidate genes in the pangenome for breeding work, as it can avoid the disadvantage of having incomplete information on a single reference genome. Among the identified *BnLTPs*, 22 and 339 pairs were tandem duplicates and segmental duplicates, respectively, it indicated that the gene duplications played an important role in the expansion and evolution of *nsLTP* residence in rapeseed. This phenomenon is not only found in rape, but also in other species, e.g., *A. thaliana*, turnip, rice, and maize [2, 6, 47]. In this study, the phylogenetic relationships of *nsLTPs* between *B. napus* and the *A. thaliana* showed a high degree of consistency in the same type between the two species.

Previous studies have shown that the *nsLTPs* are involved in a variety of biotic and abiotic stress responses [5, 48] .Odintsova et al. speculated that *nsLTPs* are involved in the activation of pathogen defences [5]. Their mechanisms of action might be due either directly to the antimicrobial or lipid-transferring activity or indirectly as signalling molecules [5]. Another study found that overexpression of *AtLTP4.4* could protect against the oxidative stress that induced by trichothecene by increasing the glutathione-based antioxidant defence effect [49]. These results suggested that the *nsLTP* family might play an important biological role in plant responses to pathogen infection. In order to study the role of *BnLTP* in the response to *S. sclerotiorum*, we analyzed the sequencing data (accession no. SRP053361) of two rape varieties (a resistant line and a susceptible line) under *S. sclerotiorum* infection for 24, 48, and 96 h [43], it was revealed that the sixty-one *BnLTPs* were identified to be differentially expressed in different samples, which also indicated that different *BnLTP* types have different expression patterns (Fig. 7D).

The experiment in vitro showed that *nsLTPs* are involved in many plant disease resistance**.** In some in vitro studies, heterologous expression and purification of nsLTPs showed resistance to a variety of plant pathogens [50, 51]. nsLTP is a plant disease-related protein (PRs) and the expression level increased with the inducing of pathogenic bacteria as well as inhibit the reproduction of pathogenic bacteria and induce the resistance-related response in plants [51]. By some biochemical methods and overexpression or knockout experiments, it was demonstrated that some nsLTPs are playing important role in resisting the invasion of pathogens [52–54]. For example, Patar et al. overexpressed the onion (*Allium cepa*) Ace-AMP1 in rice, which improved the resistance of rice blast, sheath blight and parabola without changing other agronomic traits [50]. Overexpressed wheat Ltp 3F1 in tobacco improved plant resistance to fungal diseases [51]. Inhibiting the expression of CALTPI and CALTPII in pepper, transgenic plants showed susceptibility to bacterial diseases. After overexpressing CALTPI and CALTPII in tobacco, disease resistance improved and the degree of disease resistance increased with the expression level of CALTPI and CALTPII [55]. nsLTPs were reported to have a certain role in plant disease resistance signal pathways. Maldonado et al. found that Arabidopsis nsLTP (DIR1) was involved in the process of an important mobile signal production or transmission in leaf which was infected with Phytophthora syringae [56]. Furthermore, they proposed that DIR1 interacts with a lipid-derived molecule to promote long-distance signal transmission. Grafting wild type tobacco and CALTPI and CALTPII overexpressed tobacco showed that the CALTP participates in long-distance system signaling transmission and plays an important role in the interaction of plant pathogens by activating SAR-mediated signaling pathway [55]. The above studies showed that nsLTPs play an important role in the resistance of plants and the invasion of a variety of pathogens. To further assess possible disease resistance-related functions of *BnLTPs*, we compared the position of *BnLTPs* with known QTL for blackleg resistance [44]. The blackleg resistance QTLs contained 14 core and variable BnLTP genes. Two genes (*BnLTPG.10* and *BnLTPG.12*) showed the lowest mutation in the LepR1 locus but had a large number of missense variants, it may be indicative that these genes were under positive selection pressure (Fig. 8B). There was a more synonymous variants that occurred on LepR1 locus compared with Rlm3 and Rlm4 loci, revealing that LepR1 locus is more stable than Rlm3 and Rlm4 loci. Identification of *BnLTPs* within QTL may provide a basis for *Brassica napus* breeding in the future. According to previous studies, CNVs (copy number variations) are major genetic variations influencing gene expression and phenotype. There are more CNVs than chromosome structural variations, and the total number of nucleotides covered by CNVs is much larger than SNP number in the whole genome [57, 58]. Therefore, in the future research, we will analyze the impact of CNVs on *nsLTP* gene families. The characteristics and expression patterns of *BnLTPs* identified in this study provide more information on resistant materials for molecular breeding.

## Supplementary Information

**Additional file 1: Supplymentary file 1**: The nsLTP protein set with classifications

**Additional file 2: Supplymentary Table S1**: Count of *BnLTP* genes in *Brassica napus* pangenome

**Additional file 3: Supplymentary Table S2**: Ka/Ks analysis of tandem duplication *BnLTP* genes

**Additional file 4: Supplymentary Table S3**: Ka/Ks analysis of segmental duplication *BnLTP* genes

**Additional file 5: Supplymentary Table S4**: The differentially expression analysis results of BnLTP genes that responses to Sclerotinia sclerotiorum

## Data Availability

The data presented in this study are available on request from the corresponding author. All databases used in the study are open for public access. The references of these databases are as follow: the *B. napus* pangenome information resource (http://cbi.hzau.edu.cn/bnapus/) [22]; TAIR (http://www.arabidopsis.org); NCBI (https://www.ncbi.nlm.nih.gov); Binding site prediction tool (http://plantregmap.gao-lab.org/binding_site_prediction.php in plantregmap); Retrieve mapping tool (https://www.uniprot.org/uploadlists/);AgriGO (http://bioinfo.cau.edu.cn/agriGO/analysis.php); bedtools v2.16.2 intersect [33]; Known blackleg resistance-linked QTL were collected from the literature [34–38]. Waterfall plots were drawn using Variant Effect Predictor v99 [39], GenVisR v1.11.3 [40], vcftools v0.1.16 [41].

## Abbreviations

nsLTPs: Non-specific lipid transfer proteinsQTL: quantitative trait locus
ECM: 8-cysteine motif
LTPs: Lipid transfer proteins
GPI: Glycosylphosphatidylinositol
DRN1: Disease-related non-specific lipid transfer protein 1
CDS: Coding sequence
CDD: Conserved domain database
GG: Gangan
ZY: Zheyou7
SL: Shengli
TA: Tapidor
QU: Quinta
WT: Westar
NO: No2127
*B. napus*: *Brassica napus*
*B. oleracea*: *Brassica oleracea*
*B. rapa*: *Brassica rapa*
ERF: Ethylene-responsive factor
NAC: NAM, ATAF, and CUC
LBD: Lateral organ boundaries domain
R-line: Resistant line
S-line: Susceptible line
SNPs: Single nucleotide polymorphisms
PAV: Presence-absence variation
RGA: Resistance gene analog
*S. sclerotiorum*: *Sclerotinia sclerotiorum*
