# Supplementary material for: Characterization of non-specific lipid transfer protein (nsLtp) gene families in the Brassica napus pangenome reveals abundance variation
Source: BMC Plant Biol. 2022 Jan 7;22:21. doi: 10.1186/s12870-021-03408-5 (PMC8740461; doi:10.1186/s12870-021-03408-5)
Supplement: Supplementary file 1 — Additional file 1: Supplymentary file 1: The nsLTP protein set with classifications [file 12870_2021_3408_MOESM1_ESM.docx]

>BnLTP1.1

MGNITKNQTMLLLVVTLLMVIAYHEGEAIQCSQITMYLAPCLSYVKGGGNPPPPCCAGLNNLKSSAPGRPDKQAACQCLKNVANAISGFNDDNAKQLPAKCGVSVGVPFSKSVDCNRKKCNSFFLVLNEAQILSLAFQGKFIPKFLALSQVFISPHSTFTNC

>BnLTP1.2

MRSLLLALFLVLAFHRGEAAVSCNAVVGDLYPCLSYVVQGGNVPANCCNGIRTLNSQAQTPVDRQGVCRCIKNAIGGVSFSSNNLNNAQSLPAKCGVNLPYSISPSTNCDSIN

>BnLTP1.3

MEGFIKLSTLLIVCMLVSAPMAEAAISCGAVASNLGQCINYLTRGGFVPRGCCSGVQRLHSMARTTRDRQQACRCIQGAARALGSRLNPGRAARLPGACRVRISYPISARTNCNK

>BnLTP1.4

MTFASKIITCLLVLTIYIAAPTESHITCGTVTSTMTQCISYLTNGGPLPSSCCVAVKSLNQMAQTTPDRRQVCECLKSAGKEIKGLNIDLVAALPTTCGVSLSYPIGFNTNCDRYLSIYNFVFGFILDRFASF

>BnLTP1.5

MAIALRFFTCLVLTVCIVASVDAAITCGTVTSSLAPCATYLSSGGEVPPPCCAGVKKLNGMAQTTADRQQACKCLKAAAQGINPSLASSLPGKCSVSIPYPISMSTNCDK

>BnLTP1.6

MTMGLKFFTCLVLTVCIAASVDAALTCGTVTSSLAPCATYLSKGGAVVPGPCCAGVKKLNDMAQTTPDRQQACKCLKAAAKSINPSLASGLPGKCSVSIPYPISMSTNCDNVK

>BnLTP1.7

MASALSFFTCLVLTVCIVASVDAAISCGTVTSNLVPCAGYLMKGGPVPASCCAGVSKLNSMAKTTPDRQQACKCLKTAAKSVNPSLASSLPGKCGVSIPYPISMSTNCNT

>BnLTP1.8

MTNITSKTWTMLLFVITLLMVIAYHEGEAISCSQVNMFLAPCLSYLRGGGNPSQPCCAGLNSLKAAAPGKAERQTACQCLKSVSNTIPGINDDNAKQLPSKCGVDLGVPFSKSVDCNRYTILQLLSFIWSKSSNTHQFILHV

>BnLTP1.9

MRSLLLALFLVLAFHRGEAAVSCNAVVGDLYPCLSYVVQGGNVPANCCNGIRTLNSQAQTPVDRQGVCRCIKNAIGGVSFSSNNVNNAQSLPAKCGVNLPYSISPSTNCDR

>BnLTP1.10

MGNITKNQTMLLLVVTLLMVIAYHEGEAIQCSQITMYLAPCLSYVKGGGNPPPPCCAGLNNLKSSAPGRPDKQAACQCLKNVANAISGFNDDNAKQLPAKCGVSVGVPFSKSVDCNRKKCNSFFLVLNEAQILSLAFQGKFIPKFLALSQVFISPHSTFTNC

>BnLTP1.11

MSILKNLVTIFVLGIFLTPRYSESAISCSVVLSDLQPCVSYLTSGSGQPPETCCDGVRSLDAATTTSADKKAACQCIKSVANSVTVKPELAKALASNCNASLPVDASSTVDCNTYV

>BnLTP1.12

MAFASKIITCLLVLKVYMAAPAESHITCGIVTSTLAQCMGYLTNFFPVPSDYCCAEVKGLNQMAQTTPDRRQVCKCLKAVAKENKGFISIELVGTLPTICGVSVPYPFNFSTNCDTYVSNTCYCFFLYYIRFPCFFLEYGKTFKSFLSKKVS

>BnLTP1.13

MEGLLKLSTLVIVCMLVSAPMASEAAISCGAVASNLGQCINYLTRGGFVPRGCCSGVRRLNSMARTTRDRQQACRCIQGAARALGSRLNPGRAARLPGACRVRIAYPISARTNCNK

>BnLTP1.14

MEGFLKLSTLLIVCMLVSAPMASEAAISCGAVASNLGQCINYLTRGGFVPRGCCSGVQRLHSMARTTRDRQQACRCIQGAARALGSRLNPGRAARLPGACRVRISYPISARTNCNK

>BnLTP1.15

MAFASKIITCLLILTIYIAAPTESHITCGTVTSTMTQCISYLTNGGPLPSSCCVAVKSLNQMAQTTPDRRQVCECLKSAGKEIKGLNIDLVAALPTTCGVSLSYPIGFNTNCDR

>BnLTP1.16

MAIALRFFTCLVLTVCIVASVDAAITCGTVTSSLAPCATYLSSGGEVPPPCCAGVKKLNGMAQTTADRQQACKCLKAAAQGINPSLASSLPGKCSVSIPYPISMSTNCDK

>BnLTP1.17

MTMGLKFFTCLVLTVCIAASVDAALTCGTVTSSLAPCATYLSKGGAVVPGPCCAGVKKLNDMAKTTPDRQQACKCLKAAAKSINPSLASGLPGKCSVSIPYPISMSTNCDNVK

>BnLTP1.18

MNRHTTTLVVLSLLLVVSDHTRLMIRVHSYVPFCAYTYDYFSYCLEFLTGNYYKPGKKCCVHIAKLNIIAKHKKENPRLLCNCVEMMTRGYTPPMLADKIQELPPLCNTHLSFPISSSMDCST

>BnLTP1.19

MASALSFFTCLVLTVCIVASVDAAISCGTVTSNLAPCAVYLMKGGPVPAPCCAGVSKLNSMAKTTPDRQQACKCLKTAAKNVNPSLASSLPGKCGVSIPYPISMSTNCDT

>BnLTP1.20

MAGLMKLACLVFACMIVAGPITSNAALSCGTVSGYVAPCIGYLAQNAPAVPRACCSGVTSLNNMARTTPDRQQACRCLVGAANSFPTLNAARAAGLPKACGVNIPYKISKTTNCNSVK

>BnLTP1.21

MRSLLLALFLVLAFHRGEAAVSCNAVVGDLYPCLSYVVQGGNVPANCCNGIRTLNSQAQTPVDRQGVCRCIKNAIGGVSFSSNNLNNAQSLPAKCGVNLPYSISPSTNCDR

>BnLTP1.22

MVFPSKITTCLLVLAVYMAAPSESTITCGTVTSTLARCIGYLTNSGSLPSDCCVGVKSLNQMAQTTPDRRQVCECLKSAAKDITGLNTDLVATLPTTCGVSVPYPIRFSTNCDTISTAV

>BnLTP1.23

MRDFTAKTMLLFVITLLVVIAYHEGEAISCSQVNMFLAPCLSYLRGGGNPSQPCCAGLNSLKAAAPGKAERQTACQCLKSVSNTIPGINDDNAKQLPSKCGVDLGVPFSKSVDCNRYILQWLSSITHQAST

>BnLTP1.24

MEGLLKLSTLVIVCMLVSAPMASEAAISCGAVASNLGQCINYLTRGGFVPRGCCSGVRRLNSMARTTRDRQQACRCIQGAARALGSRLNPGRAARLPGACRVRIAYPISARTNCNK

>BnLTP1.25

MSILKSLVTISVLGIFLTPRYSESAISCSVVLNDLQPCVSYLTSGSGKPPQTCCDGVRRLDAATTTSADKKAACQCIKSVANSVTVKPELAKALASNCNASMPVDASSTVDCNTYV

>BnLTP1.26

MAGVMKLACLVLACMIVAGPITANAALTCGTVNSNVAPCIGYITQGGTLPGACCTGVSKLNSMARTTPDRQQACRCLETAARALGPNLNAGRAAGIPKACGVSVPFPISTNTNCNNVK

>BnLTP1.27

MRSLLLALFLVLAFHRGEAAVSCNAVVGDLYPCLSYVVQGGNVPANCCNGIRTLNSQAQTPVDRQGVCRCIKNAIGGVSFSSNNLNNAQSLPAKCGVNLPYSISPSTNCDR

>BnLTP1.28

MVSALRFFTCLVLAVCIVASVDAAISCNTVVGSLGTCYVYLRQGGIVPPSCCRGVRNLNGMAQTTPDRQQACKCVQSFTKSVPGLNQNLASGLPGKCGVSLPYPISISMNCDK

>BnLTP1.29

MVFPSQITTCLLVLAVYMAAPSESTITCGTVTSTLARCIGYLTNSGSLPSDCCVGVKSLNQMAQTTPDRRQVCECLKSAAKDITGLNTDLVATLPTTCGVSYIDCRVKEARDVRLIKVASF

>BnLTP2.1

MVKVMWVFVLALVAALLLVTVEKIPVAEGVTCSVTELSPCLAAFMSSSQPSASCCAKLREQKPCLCGYMRNPSLRQYVTSPNAKKVSNSCKVASPHC

>BnLTP2.2

MVKVMWVFVLALVAALLLVTVEKIPVAEGVTCSVTELSPCLAAFMSSSQPSASCCAKLREQKPCLCGYMRNPSLRQYVTSPNAKKVSNSCKVASPHC

>BnLTP2.3

MKLTAIGLVAMVTIVVQLSPTMACDVKDLSPCLLPIAVFPESPTAACCQTLRDQGPCLCVFINNSWIWIGPTLTSPNGHKLFAACQVPFPSCGN

>BnLTP2.4

MKLTAIGLIAMVAIVMQLSPTMACDVIDLQPCLLPIINPPEPPTAGCCQALRDQGPCMCYFIKNTWIGPTIQAPNGHKLFADCNVPYPSC

>BnLTP2.5

MLMVKATWVSILALAVFLLVVLVPAAEAVTCSPMQLSPCAAAITSSSPPSALCCAKLKEQKPCICGYMRNPSLRRYISSPNARKVSNTCKFPMPRC

>BnLTP2.6

MKLTAIGLVAMVTTVMQLSPTMACDVIDLQPCLLPIINPPEPPTASCCQALRDQGPCMCYFIKNTWIGPTIQAPNGHKLFADCNVPYPSC

>BnLTP2.7

MEMIKAKWVSIVALAAIFLVVILVPAAEAVTCSPMQLSPCASAITSSSQPSALCCAKLKEQKPCLCGYMRNRSLRRFVSSPNARKVSNRCKLPIPRC

>BnLTP2.8

MEMIKAKWVSIVALAAIFLVVILVPAAEAVTCSPMQLSPCASAITSSSQPSALCCAKLKEQKPCLCGYMRNRSLRRFVSSPNARKVSNRCKLPIPRC

>BnLTP2.9

MKIMVLTLMVFVILLTLFPAPNEAADTNVEAACDPKQLQPCLAAITGGGQPSGDCCAKLKEQQPCLCGFSKNPAFAQYISSPNSRKVLTACGIPYPSC

>BnLTP2.10

MKFSCSKPVLFTCAILLLLIVAQENRVVAGQSCDPMQLIPCEEAILKGSKPSDTCCTRLNQQQHCVCQYMKNPNFKSFLDSPNAKKIATDCHCPKPKC

>BnLTP2.11

MKFTGVVCIAFVIVLVSASAPTKEVLEEKVACNSTEHITCIPALQSGSQPSAECCGKLKEEESCLCGYIQNPLFSQYVTSANVHMVLVTCGIPYPSC

>BnLTP2.12

MKSTTLLVFIAFVVVLVSSPPQIESRVVVESTKPPCTKIDLTGCWPELFDGTKPSAQCCGTLKAQQPCYCDFIKNPTLRKFATSPEAHLALGFCGIPFPTC

>BnLTP2.13

MKFTTLASIAFVVVVLFSSTAAPINSQLIQSNSPCTTIDITGCVPAILYGAPLSPECCRNLNVQQPCYCDFIKNAGLKPYITSPQGHAALASCGIPYPTC

>BnLTP2.14

MKSTTLVVFIVFVVVGLVSSPPQIESRVVVESTKPPCTKIDLTGCWPELFDGTKPSAQCCGTLKAQQPCYCDFIKNPALKKFATSREAHLALGFCGIPYPTC

>BnLTP2.15

MLMVKATWVSILALAAFLLVVLVPAAEAVTCSPMQLSPCAAAITSSSPPSALCCAKLKEQKPCICGYMRNPSLRRYISSPNARKVSNTCKFPMPRC

>BnLTP2.16

MKFTTLMVITLVIIAMSSPVPIRATSVESFGEVAQSCVVTELAPCLPAMTTAGDPTTECCDKLVEQKPCLCGYIRNPAYSMYVTSPNGRKVLDFCKVPFPSC

>BnLTP2.17

MVMIKATWVSIFAIAAVLLVILAPAAEAVTCSPMQLSPCAQAITSSSPPSALCCAKLKEQKPCLCGYMRNPSLRRFVSSPNARKVSNRCKLPIPRC

>BnLTP2.18

MKFTGAICIAFVLIFVSSLARTNAVVEEGTNGVCVPGELKPCIPAVQTGSKPTTECCGVLKKQQSCLCGYIKDPRFGQYVKSKYAHMVLSTCGVPYPKC

>BnLTP2.19

MKFTGAICISFVIALVSSLAWTDATVEEGIKVACVPAELKPCTPAGLTGSNPSTECCGKLKEQESCLCSYMKNPAFGHCFKTPNAHKVIAACSVAYPAC

>BnLTP2.20

MKLTAIGLVAMVTIVVQLSPTMACDVKDLSPCLLPIAVFPESPTAACCQTLRDQGPCLCVFINNSWIWIGPTLTSPNGHKLFAACQVPFPSCGN

>BnLTP2.21

MKLTAIGLIAMVAIVMQLSPTMACDVIDLQPCLLPIINPPEPPTAGCCQALRDQGPCMCYFIKNTWIGPTIQAPNGHKLFADCNVPYPSC

>BnLTP2.22

MKILALTLMVFVILSPSFAAPTKVALGAACDAKQLQPCLAAITGGGQPSGDCCAKLKEQQPCLCGFSKNPAFAQYVSSPNSRKVVSDCGVPYPSC

>BnLTP2.23

MKAYSTKPVLITCTILLLLIVAQENRVAAAEQCNPMQLMPCEDAIMKGSIPSNECCTRLNHQQHCICQYMKNPNFKSFLNSPNAKMVASHCHCKPKC

>BnLTP2.24

MVKMMWGSSLALAAALLLVTVANIPVAEGVTCSPTELTSCSSAFMSASPPSATCCAKLREQKPCLCGYLRNPALSQYVNSPNAKKVASSCNVATPKC

>BnLTP2.25

MRFTGVVCIAFVIVLVSALAPTKADLEEKVACIPTELMTCIPALQTGSQPSAECCGKLKEQESCLCGYIQNPLFSQYVTSENAHKILATCGIPYPTC

>BnLTP2.26

MCWNPIVSMKFTGVVCIAFVIVLLSALAPTKAVFEEKVACIPTELMTCIPALQTGSQPSADCCGKLKEQESCLCGYIQNPLFSQYVTSENAHKVLATCGISYPTC

>BnLTP2.27

MKMVALLLITFVIALASFPPPTSSKSNAKSCLTSAVKVCFDGIARGAPVKPACCASLKEHHTCLCDIIKSRMVDTSVLSSSLKSCGMPNPKC

>BnLTP2.28

MKFTGAICIALVIVLVSSLDLTSAAVEEEIKVACVVTELIPCLESSIIGVHPYPECCVTLKAQQSCLCGYIQNPVYGGFFKNAHSVFTGCGVPYPTC

>BnLTP2.29

MKFTRAICIALVIVLVSSLDLTSAAVEEEIKVACVQTELIPCFVAAFIGSQPSAECCEKLKEQQSCLCGYISNPVFGQFYKNAQNVFKACGVPYPTC

>BnLTP2.30

MEMIKASWVSIFAFAAILLVIIVPAAEAVTCSPMQLSPCASAITSSSQPSALCCAKLKEQKPCLCGYMRNRSLRRFVSSPNARKVSNRCKLPIPRC

>BnLTP2.31

MKIMVLTLMVFVILLTSFPVPNKAADTNVEAACDPKQLQPCLAAITGGGQPSGDCCAKLKEQQPCLCGFAKNPAFAQYISSPNSRKVLTACGIPYPSC

>BnLTP2.32

MKFSCSKPALFTCAILLLLIVAQENRVVAGQSCDPMQLIPCEEAILKGSKPSDTCCTRLNQQQHCVCQYMKNPNFKSFLDSPNAKKIATDCHCPKPKC

>BnLTP2.33

MKSTTLVVFIAFVVVLVSSPPQIESRVVVESTKPPCTTIDLTGCWPELFDKTKPSAQCCGTLKAQQPCYCDFINNPALRKFATSPEAHLALGFCGIPFPTC

>BnLTP2.34

MVMVKATWVSILALAAFLLVVLVPAAEAVTCSPMQLSPCAAAITSSSPPSALCCAKLKEQTPCICGYMRNPSLRRYISSPNARKVSNTCKFPMPRC

>BnLTP2.35

MKFTGAICMAFLIVLVLSLALTNAAMEDEKVLSCNPKELSPCSPAVKTGSKPSTECCAMLKKEEPCLCGYVNDPGYGQYIKSKNAHKAFSACGIPPLSC

>BnLTP2.36

MKFTGAICIAFVILFVSSLARTNAVVEEGTNGVCVPGELKPCIPAVQTGSKPTTECCGVLKKQQSCLCGYIKDPRFGQYVKSKYAHMVLSTCGVPYPKC

>BnLTP2.37

MKFTGAICISFVIALVSSLAWTNATVEEGIKVACVPAELKPCTPAGLTGGTPSTECCGKLKEQESCLCSYMKNPAFGHCFKTPNAHKVIAACSVAYPAC

>BnLTP2.38

MVKVMWVSVLALVAALLLVTVEKIPVAEGVTCSVTELSPCLAAFMSSSPPSASCCAKLREQKPCLCGYMRNPSLRQYVTSPNAKKVSNSCKVASPHC

>BnLTP2.39

MVMIKATWVSIFAIVAVLLVILAPAAEAVTCSPMQLSPCAQAITSSSPPSALCCAKLKEQKPCLCGYMRNPSLRRFVSSPNARKVSNRCKLPIPRC

>BnLTP2.40

MKASCTKLVLITCTLLLLLIVAQENRVAAAEQCNPMQLMPCEDAIMKGSTPSNECCTRLNQQQHCICQYMKNPEFKSFLNSPNAKMVASHCHCKPKC

>BnLTP2.41

MKLTAIGLVAMVTIVMQLSPTMACDVIDLQPCLLPIINPPEPPTASCCQALRDQGPCMCYFIKNTWIGPTIQAPNGHKLFADCNVPYPSC

>BnLTP2.42

MKLTAIGLVAMMTIVVQLSPTMACDVKDLFPCLLPITNLPEPPTAACCQTLRDQGPCLCVFINNSWLWIGPTLTSPNGHKLFAACQVPFPSCGN

>BnLTP2.43

MKLTAIGLVAMVTIVVQLSPTMACDVKDLSPCLIPIAKLPEPPTAACCQILRDQWPCLCVLINDSRLWIGHTLTSPNGHKLFAACQVPFPSCGN

>BnLTP2.44

MCWNPIINMKFTGVVCIAFVIVLLSALAPTKAVFEEKVACIPTELMTCIPALQTGSQPSAECCGKLKEQESCLCGYIQNPLFSQYVTSENAHKVLATCGIPYPTC

>BnLTP2.45

MRFTEVVCIAFMIVLVSALAPTKAYLEEKVACIPTELMTCIPALQTGSQPSAECCGKLKEQESCLCGYIQNPLFSQYVTSENAHKVLATCGIPYPTC

>BnLTP2.46

MVKVMWGSSLALAVALLLVTVANIPVAEGVTCSPTELTSCSPAFMSASPPSATCCAKLREQKPCLCGYLRNPTLSQYVNSPNAKKVASSCNVATPKC

>BnLTP2.47

MKMVALLLITFVIALASFPPPTSSKSNAKSCLTSAVKVCFDGIARGAPVKPACCASLKEHHTCLCDIIKSRMVDTSVLSSSLKSCGMPNPKC

>BnLTP2.48

MKIVAVLLIAFVVLLASFPSPTKAIAVKGSRSKRNGESCATNALQVCFDGIAKRMMMSTTCCEQLKEHHSCLCDVIKTRILDSNVLSIYLKSCGITDPKC

>BnLTP2.49

MKFTRAICIALVIVLVSSLDLTSAAVEEEIKVACVQTELIPCFVAAFIGSQPSAECCGKLKEQQSCLCGYIQNPTFGQFIKNSQNVFTACGVPYPTC

>BnLTP2.50

MKIVALLLIAFVVLLASFPPPTKAIAVKGSRSKRNGEPCATNALQVCFDGIAKRMMMSTACCEQLKEHHSCLCDVIKTRILDSNVLSIYLKSCGITDPKC

>BnLTPC.1

MALSSSSSKSSLAMIMKVAALVALVLVATEVDGQSCNRHLSGLNVCGEFVVPGADKTNPSAECCSALEAVPSECLCNTMRIASRLPTRCSIPTLSCS

>BnLTPC.2

MVSISSSSKSSTIMKVVVMVAVVLVATVVDGQSCNTHLSGLNVCGEFVVPGADTTNPSAECCNALEAVPSDCICNTFRIASRLPTRCNIPTLSCN

>BnLTPC.3

MASSSSSSKSSLAMIMKVAALAVALVLVATEVDGQSCNRHLSGLNVCGEFVVPGADKTNPSAECCSALEAVPSECLCNTMRIASRLPTRCSIPTLSCS

>BnLTPC.4

MVSISSSSKSSIIMKVVVMVAVVLVATEVDGQSCNTHLSGLNVCGEFVVPGADTTNPSAECCNALEAVPSDCICNTFRIASRLPTRCNIPTLSCN

>BnLTPD.1

MEPNTKLVLITLVLALTLTAATGEFCGMSVSDLYSCKPYVQSKNPVTSAIDPKGPCCTALSKADFQCLCKQKTKTNPFLSSIDLDLASKLPEKCGLSGATC

>BnLTPD.2

MEPNTKLVFITLVLALTLTAATGEFCGKSVADLYSCKPYVQTKNPLTTRIDPKGSCCSALANADVQCLCKQKTKTNPFLSGINLDLASKLPDKCGLSKATC

>BnLTPD.3

MEPNTKLVVITLVLALTLTAATGEFCGMSVADLYSCKPYVQSKNPVTAAIDPKGPCCTALSKADFQCLCKQKTKTNPFLSSIDLDLASKLPEKCGLSGATC

>BnLTPD.4

MAIGYHVLTGIVMILVISGELVPGKGTCQGDIQGLMRECAVYVQRPGPKVNPSAACCKVVKRSDIHCACGRITASVQKMIDMDKVVHVTAFCGKPLALGTKCGSKSITS

>BnLTPD.5

MAIGSHVLTGIVMILFISGELIVPGKGTCQGDIEGLMRQCAVFVQRPGPKVNPSAACCKAVKKSDILCACGRITSSVHNAKIINMEKVVHVTGFCGKPFAHDTKCGSKSIGS

>BnLTPD.6

MAIGSHVLTGIVMILFISGELIVPGKGTCQGDIEGLMRQCAVFVQRPGPKVNPSAACCKAVKKSDILCACGRITSSVHNAKIINMEKVVHVTGFCGKPFAHDTKCGSKSIGS

>BnLTPD.7

MASGRIIIMVVAIAFFMIGSDNVNVATAQFCGANVSGLMNECQRYVSNAGPNSPPPSRSCCALIRPIDVPCACRYVSRDVTNYIDMDKVVYVARSCGKKIPSGYKCGSYTIPAA

>BnLTPD.8

MGSGMITVMVVAIAFFMIGSDNVNMATAQLCGANLSGLVNECQRYVSNAGPNSPPPSRSCCALIRPIDIPCGCRYVTRDVMNTFDMDKLIYVARSCGKKIPSGYKCGSYTIPAA

>BnLTPD.9

MASKKVGVMVMMMMIVVVMAIFAERSVAIDLCGMTQSELNECKPAVSKENPTNPSTLCCDYLKHADISCLCGYKNSPLLGSFGIDPALAAGLPTKCDMPNAPTC

>BnLTPD.10

MGKNNTTILIIAMVLTTAMIMEEAKSYPICNTDTNDLQKCSPAVTGNSPPAPGPDCCAVVKSADLECLCPYLSRSGIDPSKIKSVLASCGVGNPSCLS

>BnLTPD.11

MATGSRVLIGLAMILIISGELLVPGQGTCQGDIEGLMRECAVYVQRPGPKVNPSAACCKVVKRSDIPCACGRITPSVQKMIDMNKVVLVTSFCGRPLAHGTKCGSKSIINQAASYFQ

>BnLTPD.12

MGKNNARILITVLAMVLTAAMMIQESKSIPLCKVNTNDLQKCRPAVTGNYPPPPTPACCTVAKTANLECLCPFLSRSGIDPAKLKALFANCGVNNPSCLPWSVNTKVPLTIL

>BnLTPD.13

MGKNNTNILTQSTVLAMVLTAAIMVKEVSSLTICKIDINDMQKCRPAVIGINPPPPVNECCVVVRSANLECFCGFKFYLPILGIDPSKVAALVAKCGVTTIPPSCQVSKVLASRSLKNGA

>BnLTPD.14

MSSSYILCITLLIFKSPIFLQLVLAQVPATCASMLLSLAPCGPFVQGFVQLPAQPCCNGLNQIYSQQPSCLCLLLNNTSTVSPAFPINQTLALQLAPLCNIPANSSSCSSLVLEAPSNSSSVAPPPSSSTGSQVSIGAKNNSTIATPVAPRPTSFLGLGYGLRSSGFKSEIQLNLSEY

>BnLTPD.15

MASKKVGVMVMMMMIVVVMAIFAERSVAIDLCGMTQSELNECKPAVSKENPTNPSTLCCDYLKHADISCLCGYKNSPLLGSFGIDPALAAGLPTKCDMPNAPTC

>BnLTPD.16

MGKNNTTILIIAMVLTTAMIMEEAKSYPICNTDTNDLQKCSPAVTGNNPPAPGPDCCAVAKSADLECLCPYLSLSGIDPSKIKSVLASCGVGNPSCLS

>BnLTPD.17

MGKKDTRILMRFAVLTMVLTAAIMVKEVTSLTLCKIDSNDMEKCRPAVTGNNPPPPVNECCVVVKSADLACFCRYKFYLPILGIDPSKVAALVAKCGVTTIPRNCRGINTYI

>BnLTPD.18

MMMRPMRVGLAMTLLMTITVLTIATAQQEDQQPPPPMLPVEEVGMCSRTFFSALVQLIPCRAAVAPFSPIPPTESCCSAVVTLGRPCLCLLANGPPLSGIDRSMALQLPQRCFANFPPCDVIN

>BnLTPD.19

MNNSHVLKTVILIMVLVESGLVEEATAHPCGRTFLSALIQLVPCRPSVAPFSTLSPNGLCCAAIKRLGQPCLCALSKGPPISGVDRALSLQLPGKCSANFPPCN

>BnLTPD.20

MGSGMTTVMVVAIAFFMIGSDNVNMATAQLCGANLSGLVNECQRYVSNAGPNSPPPSRSCCALIRPIDIPCGCRYVTRDVTNAFDMDKLIYVARSCGKKIPSGYKCGSKYYLNQVY

>BnLTPD.21

MAIGSHVLTGIVMILFISGELIVPGKGTCQGDIEGLMRQCAVFVQRPGPKVNPSAACCKAVKKSDILCACGRITSSVHNAKIINMEKVVHVTGFCGKPFAHDTKCGSKSIGS

>BnLTPD.22

MGKNNTKILITALVMVVTAAMMIEEATSIPICGVNTNDLKKCSPAVTGNNPPPPTPQCCKVAKAANLECLCPYFTRSGLDPAKIKALGTNCGITKKPSCLPW

>BnLTPD.23

MGKNNTKILITALVMIVTASMMIEEAKSVRICNVSTKDLKKCRPAVTGNNPPPPTPQCCQVAKAANLECLCPFLSRSGIDPSKIKALGANCGITKNPSCLPW

>BnLTPD.24

MDENNTRTIVAALVIVFVSLVLMEEPTSIPLCNINANTLEKCRPAVTGNNPPLPGDACCIVLQAADLECVCKFKSHIPILATKSHKVHDLLRKCGIKTIPPACQDKTKVS

>BnLTPD.25

MKEQIFCQFLVVCMLLWSSQIQGNRCDDSGIQALMACGYSIAKELPSPPKPLDGCCTAVRIIGMKCVCEIINKEIESAIDMQKLVNVASACGRPLVPHSQCGSKS

>BnLTPD.26

MATGSRVLIGLAMILIISGELLVPGEGTCQGDIEGLMRECAVYVQRPGPKVNPSVACCKVVKRSDIPCACGRITPSVQKMIDMNKVVLVTSFCGRPLAHGTKCGSYIVP

>BnLTPD.27

MGKNNILTQSTVLAMVLTAAIMVKEVTSLTICQIDINDMQKCRPAVTGINPPPPVSECCVVVRSANLECFCRFKFYLPILGIDPSKVAALVAKCGVIAIPPSCRVSKVLASRSLKNGA

>BnLTPD.28

MSPIFRQLVLAQVPATCASMLLSLAPCGPFVQGFVQLPAQPCCNGLNQIYSQQPTCLCLLLNNTSTVSPAFPINQTLALQLAPLCNIPANSSSCSSPRVETPSNSSSVAPPPSSSTGSQVSTGAKNNSRSKATPVAPRPTSFLGLGYGLRSSGSKSEIQLIILRSNPTRNSPHLNLSEY

>BnLTPD.29

MASKKVGVMVMMMVVVVMATLAERSVAIDLCGMTQSELNECKPAVSKENPTKPSTLCCDYLKHADFSCLCGYRNSPWLGSFGIDPALALGLPSKCDMPNAPAC

>BnLTPD.30

MGKNNTTILIIAMVLTTAMIMEEAKSYPICNTDTNDLQKCSPAVTGNSPPAPGPDCCAVAMSADLECLCRYLSLSGIDPSKIKSVLASCGVGNPSCLS

>BnLTPD.31

MGKNDTRVLMRFAVLTMVLTAAITVKEVTSLTLCKIDTDDMQKCRPAVTGNNPPPPVNECCVVVKSADLACFCRYKFYLPILGIDPSKVAALVAKCGVTTIPRNCRGINTYI

>BnLTPD.32

MEESRTMLKSLILLMMISSFCLELTVAETYCHAQRRLLIDACKILIFRQAPPAECCRRIRTTPAWCVCPSVTPQRAALIDVNYAVGVIRQCGRYVARGTKCGSKCIYPLYINRG

>BnLTPD.33

MASGRIIIMVVAIAFFMIGSDNVNVATAQFCGANVSGLMNECQRYVSNAGPNSPPPSRSCCALIRPIDVPCACRYVSRDVTNYIDMDKVVYVARSCGKKIPSGYKCGSK

>BnLTPD.34

MAPPLVITVMVVAIAFFMIGSDNVNMAKAQLCGANLSGLVNECQRYVSNAGPNSPPPSRSCCALIRPIDVPCGCRYVTRDVTNDFDMDKLIYVARSCGKKIPSGYKCGSKYYLNQVY

>BnLTPD.35

MAIGYHVLTGIVMILVISGELVPGKGTCQGDIQGLMRECAVYVQRPGPKVNPSAACCKVVKRSYIHCVCGRITASVQKMIDMDKVVHVTAFCGKPLAHGTKCGSKSITS

>BnLTPD.36

MAATRNAVVFIVILAITFSSSSAVTETQAPSPPALTCTEELVMFSPCLPYVSAPPNSMSDAPDPLCCSAFSTSVNSGAGNCLCYLLRQPMILGFPFNRSRLLSLSQICSDLSSDESFESICSPSESPELPPLQSIQFTAPFVYGDRASASSPLFAISPEAAGISPTSDQPSPETDSLSSTPE

>BnLTPD.37

MLKANILTGLFLLFTLSSAQTPPAPEPVAADGPSSPTNCLVSMLNVSDCLSYVQVGSTETKPEAACCPELAGMAQSSPECVCNLLGGGASPRFGVKLDKQRAKELSSLCGVKAPSPSLCSGTFYSLRAPTFSMLLDRPIHLNSIERLQFTSSTYTFF

>BnLTPD.38

MNNSHVLKTVILIMVLVEAVLIEEATAHPCGRTFLSALIQLVPCRPSVAPFSTLSPNGLCCAAIKRLGQPCLCALSKGPPISGVDRTLSLQLPGKCSANFPPCN

>BnLTPD.39

MMMRPIRVGLAMILLMTITVLTIVTSQQEDQQPPPPMLPVEEVGMCSRTFFSALVQLIPCRAAVAPFSPIPPTESCCSAVVTLGRPCLCLLANGPPLSGIDRSMALQLPQRCFANFRPCDVIN

>BnLTPD.40

MGKNNTKILITALVMVVTAAVMIEEATSIPICGVNTNDLKKCSPAVTGNNPPPPTPQCCKVAKAANLECLCPYFTRSGLDPAKIKALGTNCGITKNPSCLPW

>BnLTPD.41

MCKNNTKILVTALVMVVTAALMIEEATSIPICGVNTNDLKKCSPAVTGNNPPPPTPQCCKVAKAANLECLCPYFTRSGLDPAKIKALGTNCGITKNPSCLPW

>BnLTPD.42

MGKNNTKILITALAMIVTASMMIEEAKSVRICNVGTKDLKKCRPAVTGNNPPPPTPQCCQVAKAANLECLCPFLSRSGIDPSKIKALGANCGITKNPSCLPWLYSKVPLKLLESG

>BnLTPD.43

MGKNNTKILITALVMVVTAAMMIEEATSIPICGVNTNDLKKCSPAVTGNNPPPPTPQCCKVAKAANLECLCPYFTRSALDPAKIKALGTNCGITKKPSCLP

>BnLTPD.44

MGKNNTKILITALVMVVTAAMMIEEATSIPICGVNTNDLKKCSPAVTGNNPPPPTPQCCKVAKAANLECLCPYFTRSALDPAKIKALGTNCGITKKPSCLP

>BnLTPD.45

MGKNNTKILITALVMIVTASMMIEEAKSVRICNVSTKDLKKCRPAVTGNNPPPPTPQCCQVAKAANLECLCPFLSRSGIDPSKIKALGANCGITKNPSCLPW

>BnLTPD.46

MAATRNAVVFIVILAITFSSSSAVTETQAPSPPALTCTEELVMFSPCLPYVSAPPNSMSDAPDPLCCSAFSTSVNSGAGNCLCYLLRQPMILGFPFNRSRLLSLSQICSDLSSDESFESICSPSESPELPPLQSIQFTAPFVYGDRASASSPLFAISPEAAGISPTSDQPSPETDSLSSTPE

>BnLTPD.47

MEESRAMLKSLILLMMISSFCLELTVAETYCHAQRRLLIDACKILIFRQAPPAECCRRIRTTPAWCVCPSVTPQRAALIDVNYAVGVIRQCGRYVARGTKCGSITVP

>BnLTPD.48

MKVMRVGIALALLITITVLTIVTAQQEDQQQPPPMLPEEVVGGCRRTFFSSLVQLIPCRAAVAPFSPVPPTESCCAAIVTLGRPCLCLLANGPPLSGIDRSMALQLPLTCSSNFPPCDVIN

>BnLTPG.5

MGMNNTRILITVLALVLTAAIMIQETKSIPLCNVDTNDLQKCRPAVTGNYPPPPTPACCTVAKTANLECLCPFLSRSGIDPSKLKALLANCGVNNPSCLPWSVKIKVPLTILETG

>BnLTPG.45

MKGLYFHLFLVTMTVVASISAATPAAPAGGGSLLDECSKDIQTVSLCLDFASGKAPNPSKKCCDAIEDIKEKDPKCLCFVIQQAKSGGQSLKDLGVQEAKLIQLPTSCQLHNASISNCPKLLGISPSSPDAAIFTSNATSTTTPAAPGGTSPATPATSSEKGGSASIKDGHAVMLLAVALMSISFLSTLPWMGLA

>BnLTPG.51

MKDLHFHIFLVTMTVIASISAATPTAPAAGGALSDECNQDFQKVTLCLDFATGKAPTPSKKCCDAIEDIKEKDPKCLCYVIQQAKTGGQALKDLGVQEAKLIQLPTACQLRNASISNCPKLLGISPSSPDAAVFTSNATTITPEAPAGKSPATPATSSEKGGSASIRDGHAVVALAITLITVSFVSTLL

>BnLTPG.58

MDPKSFLISSLIFCLLSNSPILMSLAQINTPCSPTMLSSVTGCMSFLTGGASSPTSDCCEALISLTGTGMDCLCLIVTANVPLDLPVNRTLAISLPRACGVPVQCKASSALLYAPGPASHGPTTSPPTETQYPEGPASFGPVTSPTSSMDPDGMPDDADFSGPRNGGDPREPPKTSASSPSSSLSLKLSPLLFALFAFEFISFF

>BnLTPG.83

MKQSLLSILILLLSSSFAPIHARNKPQPKSPSPVAAPAPGPSNSDCSSIIYDMMDCLSYLTPGSNDTKPTKVCCGGILSVLQYNPKCICVGLESSKTMGFAVNNTRARAMPTTCKLTIVAPHCAILDEATPAASIAVSPSAGTPMTSPSSGGSPTSSPSLAESPVMTAPSPSSSGTNHLSVSTLTLVSVIVSSVTYISFLF

>BnLTPG.103

MEIVRFAVIFVLCSISSSNAATTPPSGGAGDAHSMPCIQKLMPCQPYLHSATPPPPASCCMPLKEIVETDVNCLCSVFNNVDMLKSLNLTKENALVLPKACGANADVSQCKASTGTTTPSTSPGTTKTPPASPAESGSTGGPASSTAKPSDSAPAINFSGISFASAFVALTTILF

>BnLTPG.115

MKGLYFHLFLVTMTVVASISAATPAAPAGGGSLLDECSKDIQTVSLCLDFASGKAPNPSKKCCDAIEDIKEKDPKCLCFVIQQAKSGGQTLKDLGVQEAKLIQLPTSCQLHNASISNCPKLLGIPPSSPDAAIFTSNATSTTTPAAPGGTSPATPATSSEKGGSASIKDGHAVMLLAVALMSISFLSTLPWMGLV

>BnLTPG.49

MKQSLLSILILLLSSSFAPIHARNKSQPANSPSSVAAPAPGPSNSDCSSVIYDMMDCLSYITPGSNDTKPTKVCCGGILSVLQYNPTCVCVGLESSKTMGFAVNNTRARAMPTTCKLPIVATHCPMLDEVTPAASTPVSQSAGTPMTSPSSVASPTSSPSLAESPVMTAPSPSSSGTNHLSASTLTLVVIKVSFVAYISFFFSN

>BnLTPG.54

MDPKSFLISALIFSLLSNSPVLMSLAQINTPCSPTMLSSVTGCMSFLTGGATSTTSDCCRALKTLTGTSMDCLCMIVTANVPLDLPINRTLAISLPRACGVPVQCKASSALLYSPGPASVGPTTSPPTETQNPEGSASFGPATSPTSSMDPDGMPDDTDFSGPRNGGDPREPPKTSASSPSSSLSLKLSLLLFAMFAFEFISFF

>BnLTPG.113

MSKIPVVTIVVALLAVLALPARSQQPPLSQCTPSMMTTVGPCMSILTNSSTNGTSPSSDCCNSLRSLTTGGMGCLCLIVTGSVSFNIPINRTTAVSLPRACNMPRVPLQCNANIAPAAAPGPAGTFGPAMSPSPATTPIVPEPTPAAQTPQSDATRPFTPTVDGAAPTSDDGGRSTSRPSVTPSSSYALSPSLLFLVVSLVALKFY

>BnLTPG.114

MKTGMGLMLLTVFMAVMSSTRVSAQSSCTSALISMAPCLNYITGNTTSPSQQCCSQLRSVVQSSPDCLCQALNGGGSQLGLNINQTQALALPRACNVQTPPISRCNGGGSNADSPADSPKSSGPGNGSKTVPVGEGDGSSSDGSSIKFSYPLLAFLIAASYMAVFLKY

>BnLTPG.6

MAVFSAALPLLLLFLSVSSPSVNGNTAQSVECSAVIMTMTKCLPFVTIGSHVEKPETACCSVIKTVLDTKAECLCEGLKSSAAAGINLNLTKAGTLPDACQLKAPPMPACALFAKPPASAPAPVPAARPLNGSGPGSNSAPAPSPSHCNHGSSISVLSLAISGALVIMFTRI

>BnLTPG.110

MKQSLISILILLLSSSFVPIHARNKPQPAKSPSPVAALAPGPSNSDCSSIIYDMMDCLSYLTPGSNDTKPTKVCCGGILSVLQYNPKCICVGLESSKTMGFAVNNTRARAMPTTCKLTIVAPHCAILDEATPAASIAVTPSAGTPMTSPSSGGSPTSSPSLAESPVMTAPSPSSSGTNHLSVSTLTLVSVIVSSVTYISFLF

>BnLTPG.111

MKQSLISILILLLSSSFVPIHARNKPQPAKSPSPVAALAPGPSNSDCSSIIYDMMDCLSYLTPGSNDTKPTKVCCGGILSVLQYNPKCICVGLESSKTMGFAVNNTRARAMPTTCKLTIVAPHCGDAEKRSSDNNDAVTPSAGTPMTSPSSGGSPTSSPSLAESPVMTAPSPSSSGTNHLSVSTLTLVSVIVSSVTYISFLF

>BnLTPG.112

MKQSLISILILLLSSSFVPIHARNKPQPAKSPSPVAALAPGPSNSDCSSIIYDMMDCLSYLTPGSNDTKPTKVCCGGILSVLQYNPKCICVGLESSKTMGFAVNNTRARAMPTTCKLTIVAPHCGDAEKRSSDNNDAVTPSAGTPMTSPSSGGSPTSSPSLAESPVMTAPSPSSSGTNHLSVSTLTLVSVIVSSVTYISFLF

>BnLTPG.1

MAYTNKIPAAAAVATAMLFLAVMMAPRWTEAQTMPNLDPVCTILITDIMTKCYLSGNLAPSTECCDDLKSANSKQVTCLCDNFIAHPSNNNVTQALLEVYHHDCGVADKFVCKAGNSNGGAAKKISASIGLLGLATSLFF

>BnLTPG.2

MKPGMCLLFLTVSMAVMSTVSAQSTCTNVLISMAPCLGYITGNSSSPSQQCCSQLAHVLRSSGECLCEVLKGGGPHLGINFNKTQALALPKACNVQTPPVSLCNDDSSVKSPTGPSNTSEHGNGSKTVPGHRSTSRGSSIKVSFSLLAVLSAVSYIINYSRY

>BnLTPG.3

MTNVAVIAAILITVLLSASVSEQMAPSPSSGPSGAPDCMTNLLNMTDCLSYVQVGNGGGAANPDKACCPELAGLVDSSPQCLCYLLGGDMAAQYGIKIDKAKALKLPRVCGVVTPDPSLCSLFGIPVGAPEAMGKEEASPAFAPSSGAESPEGLGSGPSASRTSDAPNTPYSLFLSVIIIPLAFAFHLYS

>BnLTPG.4

MGRVLVLLTVFMAVMSSTRVSAQSSCTTALISMSPCLNYITGNTTSPSQQCCSQLGNVVRSSPDCLCQALNGGGSQLGINVNQTQALALPRVCNVQTPPVSGCSNGGGSTADSPTDSPNSSGPVNGSKTVPVGEGDGSSSDGSSIKISYRLLTFLSVASYIAIFLKY

>BnLTPG.7

MALFSAALPLLLLFLFVSSLSVNGNTAQSVECSAMIMTLTKCLPFVTIGSQVEKPETACCSVIKTVLDTKAECLCEGLKSSAAAGINLNLTKAGTLPDACQLKAPPMPACALFAKPPASAPAPVPAARPLNGSGPGSNSAPAPSPSHSNHGSSISVLSLAISGALVIMFTRI

>BnLTPG.8

MGMNNTRILITVLALVLTAAIMIQETKSIPLCNVDTNDLQKCRPAVTGNYPPPPTPACCTVAKTANLECLCPFLSRSGIDPSKLKALLANCGVNNPSCLPWSIEELRWTGHNPACCVLCHHLNIKSLSYYFLWCSIQIKSTNFEVIFKSPSLSTRTVFYTLMKTIKYCFWLLFHCVLLSWISKN

>BnLTPG.9

MDITRSLGVATVLVIIYSIQITAQMEQTEEAMRCVEKLIPCHPYINTDSPPPPWCCNPVKEIVEKDVTCLCGFLNHPDMLALINITQDDALNLISSCGASYDESLCSNSTVSSPDTSPGATTTESSSGSTTKNAALAISFLGFSFLWL

>BnLTPG.10

MKQSLLSILILLLSSSFAPIHARNKPQPANSPSPVASPAPGPSNSDCSSIIYDMMDCLSYLTPESNDTKPTKVCCRGILSVLQYNPKCICVGLESSKTMGFAVNNTRARAMPTTCKLPIVAPHCAILDEATPAASTAVSPSAGTSMTSPSSVGSPTSSPSLAESPVMTAPSPSSSGTNHLSVSTLTLVYVIVSSVMYILFF

>BnLTPG.11

MAQTTTVIFILATLLVAAAVVSGQTPPSPVAPSPTINEAMNCAAGLTVCLPAFAQGGTPSKECCTAVKTQQSCLCGFIKAPVLVVPFNITAFSALISKSCGINTDLNLCSETPAHAPLPHMTAPPSGTPKTDKDAASKPAETGLVGIVLIMISALFY

>BnLTPG.12

MKQSLILSILILLSSSFAQIHARNKSHPANPPSPVATPAPGPSNSDCSSVIFDMMDCLSYLTPGSNDTTPTKVCCGGILSVLQYNPKCVCIGLASSKDMGIALNNTRALAMPTICKLPIAAPHCAILDASRPSASTPGMSSVSPSAVTPMTPQSSAQSPTFSPSLPESPGITAPSPSSSGTNHLSVSKFTFVAVVVSYITYISAFSN

>BnLTPG.13

MATSFCTLTPFLFILLLSISSVLEAAHHHTAAPAPAVDCSMLILNMADCLSFVSAGGTEAKPASSCCNGLKTVLKTDAECLCEGFKSSASLGVTLNMTKAATLPAACKLHAPSMAACGLSAAPTMAPGLAPGGAAVAAGPNLSFLAPNPSPGNHGSSLLPFSFTTILSTMFFVLFLSRV

>BnLTPG.14

MRSLFLLALFLVLAFHHGEAAVTCNNVVGDLYPCLSYVMQGGNSPSTNCCSGVRTLNSQAQTTADRQSVCRCIKNAIGGASYSSSNLKNALSLPAKCGVNLPFSISPSTNCNR

>BnLTPG.15

MRSLFLLALFLVLAFHHGEAAVTCNNVVGDLYPCLSYVMQGGNSPSTNCCSGVRTLNSQAQTTADRQSVCRCIKNAIGGASYSSSNLKNALSLPAKCGVNLPFSISPSTNCNSIH

>BnLTPG.17

MGLMLLTIFMAVMPSTRVSAQSSCTSVLISMAPCLNYITGNTTSPSQQCCSQLSSVVQSSPDCLCQALNGGRSQLGLNINQTQALALPRACNVQTPPISRCNGGGSNADSPADSPKSSGPGNGSKTVPVGEGDGSSSDGSSIKFSYPLLAFLIAASYMAVFLKY

>BnLTPG.18

MSKIPVITIVVALLAVLALPVRSQQPPLSQCTPSMMTTVGPCMSILTNSSTNGTSPSSDCCNSLRSLTTGGMGCLCLIVTGSVPFNIPINRTTAVSLPRACNMPRVPLQCNANIAPAAAPGPAGTFGPAMSPSPATTPVVPEPTPAAQTPQSDTTRPFTPTVDGAAPTSDDGGSTSRPSVTPSSSYALSPSLLFLVVSLVALKFY

>BnLTPG.19

MASRTMETSILMIFTVVALMSGERALAVDCSSLILNMADCLSFVTNGSTVEKPEGTCCSGLKTVVRSGPECICEGFKNSASLGVTLDLAKAASLPSACKVAAPPSARCGLAVSASPPASSPEISPTAGAGAPSSSSEANAATPVPVPAGSSDASLVSVSFAFALFIALISSSFY

>BnLTPG.20

MEGITLIIVIMSSLILGGKGQQIISTPCTSSMISTFTPCLNFITGSSGGSVTPTAGCCDSLKSLTSTGMNCACLILTANVPLPTGFINRTLSLALPRACKMTGVPVQCQAAGTPLPAPGPVPFLLAPPPPMSAFSPGSSKAAATAPGLAPDAPLDGPMGPTATPGIRPVVQPLQPTSLAQYSTSPFLPLLFFLFTLLTLLNL

>BnLTPG.21

MESWRISLVAIAIALLMATLVSAGEDKAKDKEECTEQLVGMATCLPYVQGQAKTPTPDCCSGLKQVLKSDKKCLCVIIQDRNDPDLGLQINVSLALALPSVCHAVADVTKCPALLHMDPKSQEAQVFYQLANGLNKTGPASAPIISAPAPTSVSPTAGSEDGNNSGRATSLPSKNHAQSFRKRWLVLEVAAYLFIISFTITL

>BnLTPG.22

MAATSNAVVLIVILAITFSSSSAVTETQAPSPLALTCTEELVMFSPCLPYVSAPPNNISDAPDPLCCSAFSTSVHSGAGNCLCYLLRQPMILGFPLDRSRLLSLSQICSDLSSDESFESICSPSESPELPPLQSIQFTAPFVYGVRASASSPSFAISPEAAGISHTSDQPSPETDSLSSTPESIINGSPKIRSFWFLSTIIMTLPTSIFTRI

>BnLTPG.23

MAATSNAVVFIVILAITFSSSSAVTETQAPSPLALTCTEELVMFSPCLPYVSAPPNNISDAPDPLCCSAFSTSVNSGAGNCLCYLLRQPMILGFPLDRSRLLSLSQICSDLSSDESFESICSPSESPELPPLQSIQFTAPFVYGDRASASSPSFAISREAAGISPTSDQPSPETDSLSSTPESIINGSPKITSFCFLSTIIMTLPTSIFTRI

>BnLTPG.24

MVKVMWVSVLALVAALLLVTVEKIPVAEGVTCSVTELSPCLTAFMSSSQPSASCCAKLREQKPCLCGYMRNPSLRQYVTSPNAKKVSDSCKVASPNC

>BnLTPG.25

MKMGMGLVVLTVFMAVMSSTRVLAQSTCTSALISMSPCLNYITGNTTSPSQQCCSQLGNVVRSSPDCLCQVLNGGGSQLGINVNQTQALALPRACNVQTPPVSRCNNGGGSTADSPADSPNSSGPGNGSKTVPVGEGEGEGPSSDGSSIKFSYPLLAFLSAASYMAIFLKY

>BnLTPG.26

MEQSTRSLIITIVITSMLVGFGSSDLDQDREECTNQLVVLSPCLTYVGGNAKAPTKDCCGGFGQVITQSQKCVCILVKDKDDPNLGLKFNASLAAHLPTACHITAPNITKCISLLHLSPNSTLAREFESLGRLEASANSAPPLQNVKDGAGGGKAESVKSDGGKKKKSWLAVELLIFALFSHLLLVISSFTSSSFI

>BnLTPG.27

MEVVRFAVAVVFVLFSVSSSNAEPTPAMGGGGGGGGGDAHSMPCIQKLMPCQPYLHSVTPPPPASCCLPMKEIVEKDATCLCSVFNNVDMLKSLNLTKENALVLPKACGAKADISLCKSSNGTTTPSTGTTTTPPASSTGSGSTGASSSSTAKPTNSAPGITFAGASFASAFMALATIFF

>BnLTPG.28

MASSSVFITVLISLVPVFLQPGLAQGQSPPASCASLLLALAPCGPFVQGFVQFPAQPCCSSLSQIYSQQPTCLCLFLNNNSTLSSAFPINQTLALKLPQLCSIPANSSVCSSGASTASPPSTNSTGSQVSMGAKNNSVAAATPVAQVAPKPSNMMGLGDGLRSSGPKSKIQVTIFVIAAILAGTLFLV

>BnLTPG.29

MATSFSAATPFVFILLLSISSVTVHGASHHHTAAPAPAVDCSTLIINMADCLDFVTAGGTSAKPKSSCCAGLKTVLKADAECLCEAFKNSAAFGITLNMTKAATLPTACKLHAPSISNCGCKICLAPGGAVAAGPGAAGTTLAPTPSQGNDGSCLIPISLTTLFSALFFVLFLSRV

>BnLTPG.31

MKGLHFHLFLVTMTVVVSVSAATPAAPAAGGALADECSQDFQKVTLCLDFATGKAPNPSKKCCDAIEDTKERDPKCLCYVIQQAKTGGQALKDLGVQEDKLIQLPTSCQLHNASISNCPKLLGISPSSPDAAVFTSNATSTTTPAAPAGKSPATPTTSMGTGGSPSIRDGHATVALAFALIMTVSFVSILPRMGLA

>BnLTPG.33

MLKANILTGLFLLFTLSSAQTPPAPEPVAADGPSSPTNCLVSMLNVSDCLSYVQVGSTETKPEAACCPELAGMAQSSPECVCNLLGGGASPRFGVKLDKQRAEELSSLCGVKAPSPSLCSVLGFPTISPAGSEDSSSAGSEGSEKDKKNGGMPTKDYGLAFNSLMLALFTIFSLY

>BnLTPG.34

MEICKFLTLIFLAIVVLYPVQATAQGGDPHLMSCMQKLMSCQPYIHAVNPPPPPSCCGPMKEIVVKDAPCLCAVFNDPAILKTLNLTKENALDLPKACGANPDISLCSKTASLPPTAPPGPTSGCSSVQAVSYIGLSFLLAFVARILY

>BnLTPG.36

MSISIFLGVSTVLAILYAVQATAIWDQDKAMVQCVAKLTPCRPYVNSEAPPPLWCCHPLRKIVENDATCLCEAFKHPDMLALIHLTQEAALNLISSCGVSYDASSCDAESSSVSTSKNAALAISLLGVSFVSAFTGL

>BnLTPG.37

MEICKFLTVIFVAVVVLYSVQAAEQGGDHHSMACMQKLMPCQNYIHAVNPAPPASCCGPMKEIVEKDSKCLCTVFNNPELLKSLNLTKENALDLPKACGVNPDVSICTKTASSPPIASPTSGGSSVQAVSIIGLAFAFAFVARILY

>BnLTPG.38

MASSILFITLLIALSSISLQLVFAQVPGTTTATCSSMIFSLAPCGPFVQGFVQLPAQPCCDGLNQIYSQQPTCLCLFLNNTSTLSPAFPINQTLALQLPPLCNVPANASSCSSPGGEAPSDSSSVAPPPSSSTGSQVSPVAKNNSATPVAQLAPRPTSLMGLGYDLRSSGSKSEIQLIILALAVILPGTLLI

>BnLTPG.39

MEKSTRTLMIITIVITSMLVGFGSSDLDQDREECTDQLIALSPCLPYVGGNAKAPTKDCCGGFDQVITKSEKCVCILVKDKDDPNLGLKFNATLAAHIPTLCHITAPNITKCISLLHLSPNSTLAKEFESLGRIEYEGKTNSTSPSHNVKDGTGGGKAEQVKSTGEKKSWLAVELLIFALFSHLLFIIPSFTSSSFI

>BnLTPG.41

MAATIVFILMLAITSSTAVAETQGPSSSPPAPTCSEELVMFSPCIPYVSAPPNNISKTPDALCCSVFSTSVHSAAGKCLCYLLRQPMILGFPLDRSRLLSLSQICTEFQSSDESFESLCSPSVSPELPPLQSIQFTSPFDYGDRDSASPQSLGLPPETAKDPPTSDQFSPDIDNVSPQLIINGSPMISNLLLFLTTIIMTLATCILTRI

>BnLTPG.43

MAYVNKVSAVAAILFFAVAVAPLLAEPQTPMFPKMDPVCASLIPNLLEKCFSTVRETPTDDCCSDLKSATTTQVTCLCDNYIANPAVVNFTGPYSAGITTKCGVFDKYSCNGSSNGGGEGSSSNSSSSSNGKDNSKSEGSGGRANKVAASIMAVFGLVASLIFVMF

>BnLTPG.44

MASGRIIIMVVAIAFFMIGSDNVNVATAQFCGANVSGLMNECQRYVSNAGPNSPPPSRSCCALIRPIDVPCACRYVSRDVTNYIDMDKVVYVARSCGKKIPSGYKCGSKYPFIFLQYFLLFVLYS

>BnLTPG.46

MKGHYFHLFLVTMTVVASISAATPAAPAGGGSLLDECSKDIQTVSLCLDFASGKAPNPSKKCCDAIEDIKEKDPKCLCFVIQQAKSGGQTLKDLGVQEAKLIQLPTSCQLHNASISNCPKLLGISPSSPAAAIFTSNATSTTTPAAPGGTSPATPATSSEKGGSASIKDGHAVMLLAVALMSISFLSTLPWMGLA

>BnLTPG.47

MEILKFAVIFVLCSISSSNAATTPPSGGAGDAHSMPCIQKLMPCQPYLRSVTPPPPASCCMPLKEIVETDVNCLCSVFNNVDMLKSLNLTKENALVLPKACGANADVSQCKASTGTTTPSTSPGTTKTPPAAPAESGSTGGSASSTAKPSDSAPAIIFSGISFASAFVALATIFF

>BnLTPG.48

MATATMIFAAAMTAMILVSLQQVEAQSATTCVNKLVPCFSALTTTTKPPKDCCDSIKEAVEDELPCLCTVYNTPGLLSQFNVSTAQALNLSRRCDVTTDLSACSGTGASSPKASLPPPAGKRGNDTAVGNKLTGYGVTTVILSLVSTIFF

>BnLTPG.50

MKQSLLSILILLLSSSFAPIHARNKSQPANSPSSVAAPAPGPSNSDCSSVIYDMMDCLSYITPGSNDTKPTKVCCGGILSVLQYNPTCVCVGLESSKTMGFAVNNTRARAMPTTCKLPIVATHCPMLDEVTPAASTPVSQSAGTPMTSPSSVASPTSSPSLAESPVMTAPSPSSSGTNHLSASTLTLVVIKVSFVAYISFFFSN

>BnLTPG.52

MKDLHFHIFLVTMTVIASISAATPTAPAAGGALSDECNQDFQKVTLCLDFATGKAPTPSKKCCDAIEDIKERDPKCLCYVIQQAKTGGQALKDLGVQEAKLIQLPTACQLHNASISNCPKLLGISPSSPDAAVFTSNATTITPEAPAGKSPATPATSSEKGGSASIRDGHAVVALAITLLTVSFVSTLPMMALA

>BnLTPG.55

MEIVRFTVAVFFVLYSVSSSNAATAPPSGGGGGGGDAQAMPCIQKLMPCQPFLHSVIPPPPPSCCLPMKAIVANDATCLCSVFNNVDMLKSLNLTKDNALDLPKACGANPDISLCKASPAGGTTTNSTSPATPKTPPVSSTGSGSTGASSSTTSPTSSAPAINFAGLSFASTIVALATTFF

>BnLTPG.56

MIGRFAVSRKTSKKFTVAVFFVLVSVSSSNAAVAPPSGGGSGGGDARALPCIQKLKSCQPYLHSVIPPLPASCCLSMKEMVANDAPCLCSVFNNVDMLKSLNLTRDNALDIPKACDAEPDISLCKASPADGPTTNSTSSTPTSSAPAINFAGLSFASTIVALATTFF

>BnLTPG.57

MEIVRFAVAAVFFVLYSVSSSNAAIAPPSGGGGGGGDAQAMPCIQKLMPCQPFLHSVIPPPPPSCCLPMKAIVANDATCLCSVFNNVDMLKSLNLTKDNALDLPKACGANPDISLCKASPAGGTTTNSTSPATPKTPPATSTGSGTTGASASSTSTPTSSAPAINFAGLSFASTIVALAATFF

>BnLTPG.59

MDPKSFLISALIFSLLSNSPILMSLAQINTPCSPTMLSSATAGCMSFLMGGASSPTSDCCRALKTLTGTSMDCLCLIVTANVPLDLPINRTLAISLPRACGVPVQCKASSALLYAPGPASLGPTTSPPTETQIPEGPASFGPATSPTSSMDPDGMPDADTDVSGPRNGGDPREPPKTSASSPSSSLSLKLSLLLFAVGTHYS

>BnLTPG.60

MDPRFCLISALIFLSLLSNSPILILAQISTPCSPTMLSSVTGCMSFLTGGGSSPTSDCCEALKSLTGTGLDCLCLIVTASVPINIPINRTLAISLPRACGMPGVPVKCKASAAPLPAPGPVSLGPTTPTETQSPQGSASFGPTTSPASSIIPDDQNIPASDKGENPTASTPSASSPSSSHSIKLPLLLLTFFAFQIISLLLS

>BnLTPG.61

MAYTNKIPAAAAVATAMLFLAVMMAPRWTEAQTMPNLDPVCTILITDIMTKCYLSGNLAPSTECCDDLKSANSKQVTCLCDNFIAHPSNNNVTQALLEVYHHDCGVADKFACKAGNSNGGAAKKISASIGLLGLATSLFF

>BnLTPG.62

MKPGMCLLFLTVSMAVMSTVSAQSSCTDVLISMAPCLGYITGNSSSPSQQCCSQLAHVIRSSGECLCEVLKGGGPHLGINFNKTQALALPKACNVQTPPVSRCNDDSSVKSPTGPSNTSEHGNGSKTVPGHRSSSRGSSIKVSFSLLAVLSAVSYIINYSRY

>BnLTPG.63

MTNVAVIAAILITGLLSASVSEQMAPSPSSGPSGAPDCMTNLLNMTDCLSYVQVGNGGGAANPDKACCPELAGLVDSSPQCLCYLLGGDMAAQYGIKIDKAKALKLPGVCGVVTPDPSLCSLFGIPVGAPEAMGKEEASPAFAPTSGAESPEGLGSGPSASKTSDAPNTPYSLFLSVIIIPLAFAFHLYS

>BnLTPG.64

MKMGRVLVLLTVFMAVMSSTRVSAQSSCTTALISMSPCLNYITGNTTSPSQQCCSQLGNVVRSSPDCLCQALNGGGSQLGINVNQTQALALPRACNVQTPPVSRCNNGGGSTTDSPTDSPNSSGPGNGSKTVPVGEGDGSHGSSIKISYRLLTFLSVASYIAIFLKY

>BnLTPG.65

MALFSAALPLLLLFLSVSSPSVSGNTAQSVECSAVIMTLTKCLPFVTIGSQVEKPETACCSVLKTVLDTKAECLCEGLKSSAAAGINLNLTKAGTLPDACQLKAPPMPACALFAKPPASAPAPVPAARPLNGSGPSSNSAPAPSPSHSNHGSSISVLSLAISGALVIMFARI

>BnLTPG.66

MGKNNARILITVLAMVLTAAMMIQEGKSIPLCKVNTNDLQKCRPAVTGNYPPPPTPACCTVAKTANLECLCPFLSRSGIDPAKLKALFANCGVNNPSCLPWSVNTNVPLTIL

>BnLTPG.67

MGKNNTRILITVLALVLTAVMMIQEAKSIPLCNVDTNDLQKCRPAVTGNHPPPPTSACCTVAKTANLECLCPFLSRSGIDPSKLKALFANCGVNNPSCLPWSIEELRWSGHKPACCVLCHHLNIRSRSYNFLWCSIQIKSTNFEVIFKSPFLSTGTVFYTLMKTIKYCFWLSSIVFYFHGYPKTNSSCLFPALD

>BnLTPG.68

MDITRSLGVATVLVILYSVQATVQMEQTEEAMRCVEKLIPCHPYINTDSPPPPWCCNPVKEIVEKDETCLCGFLNHPDMLALINISQDDALNLINSCGASYDESLCSNSTVSSPDTSPGATTTESSSGSTTENAALAISFLGFSFIWL

>BnLTPG.69

MKQSLLSILILLLSSSFAPIHARNKPQPANSPSPVASPAPGPSNSDCSSIIYDMMDCLSYLTPGSNDTKPTKVCCRGILSVLQYNPKCICVGLESSKTMGFAVNNTRARAMPTTCKLPIVAPHCAILDEATPAASTAVSPSAGTSMTSPSSVGSPTSSPSLAESPVMSAPSPSSSGTNHLSVSTLTLVSVIVSSVTYISFS

>BnLTPG.70

MAQTTTVIFILATLLVATTVVSGQTPPSPVAPSPTINEAMNCAAGLTVCLPAFAQGGTSPKECCTALETAVKTQQSCLCGFIKAPALVVPFNITAFSALISKSCGINTDLNLCSETAAQAPPLPHMTAPPPGAPKTDKDAASKPAETGLVGIALIMISALFY

>BnLTPG.71

MKQSLFLSILILLSSSFAQIHARNKSYPANPPSPVATPAPGPSNSDCSSVIYDMMDCLSYLTPGSNDTKPTKVCCGGILSVLQYNPKCVCIGLASSKDMGIALNNTRALAMPTICKLPIAAPHCAILDVSRPSASTPGMSSVSPSAVTPMTPQSSAQSPTFSPFLPESPGITAPSPSSSRTNHLSVSKFTFVAAVVSYITYISVFSN

>BnLTPG.72

MATSFPTLTPFLFILLLSIPSVLEAAHHHTAAPAPAVDCSMLILNMADCLSFVSAGGTEAKPASSCCNGLKTVLKTDAECLCEAFKSSASLGVTLNMTKAATLPAACKLHAPSMAACGCKIYYFINFFCLAPGGAAGPDLSFLSPNPSPGNRGSSLLPFSFTTVLGAMSFVLLFLSRV

>BnLTPG.73

MRSLFLLALFLVLAFHHGEAAVTCNNVVGDLYPCLSYVMQGGNAPSTNCCNGVRTLNSQAQTTADRQSVCRCIKNAIGGASYSSSNLNNALSLPAKCGVNLPFSISPSTNCNR

>BnLTPG.74

MKTGMGLMLLTVFMAVMSSTRVSAQSSCTSALISMAPCLNYITGNTTSPSQQCCSQLRSVVQSSPDCLCQALNGGGSQLGLNINQTQALALPRACNVQTPPISRCNGGGSNADSPADSPKSSGPGNGSKTVPVGEGDGSSSDGSSIKFSYPLLAFLIAASYMAVFLKY

>BnLTPG.75

MASRTMETSILMIFTVVALMSGERAIAVDCSSLILNMADCLSFVTNGSTVEKPEGTCCSGLKTVVRSGPECICEGFKNSASLGVTLDLAKAASLPSACKVAAPPSARCGLAVSASPPASAPEISPTAGAGAPSSSSEANAATPVPVPAGSSDASLVSVSFASALFIAFISSSFY

>BnLTPG.76

MATSFFTATPFVFILLLSISSVTVHGASHHHTAAPAPAVDCSTLIINMADCLDFVTAGGTSAKPKSSCCAGLKTVLKADAECLCEAFKNSASFGITLNMTKAATLPTACKLHAPSISNCGLSMTPTMAPGLAPGGAVAAGPGAAGPGAAGTTLAPTPSQGNDGSSLIPISFTTLFSALFFVLFLSRV

>BnLTPG.77

MKGLYFHLFLVTMTVVASISAATPAAPAGGGSLLDECSKDIQTVSLCLDFASGKAPNPSKKCCDAIEDIKEKDPKCLCFVIQQAKSGGQTLKDLGVQEAKLIQLPTSCQLHNASISNCPKLLGISPSSPDAAIFTSNATTTTTPVAPGGTSPATPATSSEKGGSASIKDGHAVMLLAVALMSISFLSTLPWMGLA

>BnLTPG.79

MVMIYINKVPAAVAIALLFLVVVIAPQWTKAQPLPPFPPMSPLPPLPPMSPLPPLPPMSPLPPMPKVDPVCTTAILDIVQNCYSTLSAVPSEECCNGLKSASKTQVTCLCDNFIAHPVFSNLTRPYYDQVSNACGVLDKYACNGTGEEGGDSKGGDPKGGDGNAGAINKIAGSMGLFGLITCLFFLLF

>BnLTPG.80

MGNITKNQTMLLLVFTLLMVIAYHEGEAIQCSQITMYLAPCLSYVKGGGNPPPPCCAGLNNLKASAPGKPDKQAACQCLKNVANAISGFNDDNAKQLPAKCGVSVGVPFSKSVDCNRKKCNSFFLVLNEAQILSLAFQGHKYITKFLALSQVFISPHSPTAK

>BnLTPG.81

MEKSTRTLMIITIVITSMLVGFGSSDLDQDREECTDQLIALSPCLPYVGGNAKAPTKDCCGGFDQVITKSEKCVCILVKDKDDPNLGLKFNATLAAHIPTLCHITAPNITKCISLLHLSPNSTLAKEFESLGRIEYEGKTNSTSPSHNVKDGTGGGKAEQVKSTGEKKSWLAVELLIFALFSHLLFIIPSFTSSSFI

>BnLTPG.84

MKQSLLSILILLLSSSFAPIHARNKPQPKSPSPVAAPAPGPSNSDCSSIIYDMMDCLSYLTPGSNDTKPTKVCCGGILSVLQYNPKCICVGLESSKTMGFAVNNTRARAMPTTCKLTIVAPHCAILDEATPAASIAVSPSAGTPMTSPSSGGSPTSSPSLAESPVMTAPSPSSSGTNHLSVSTLTLVSVIVSSVTYISFLF

>BnLTPG.85

MKVMRVGLALALLITITVLTIVTAQQVDQQPPPPMLPEEEVGGCSRTFFSSLVQLIPCRAAVAPFSPVPPTESCCAAIVTLGRPCLCLLANGPPLSGIDRSMALQLPLTCSANFPPCDVIT

>BnLTPG.86

MEGITLIIVIMSSLILGGKGQQIISTPCTSSMISTFTPCLNFITGSSGGSVTPTAGCCDSLKSLSSTGMNCACLILTANVPLPTGFINRTLSLALPRACKMTGVPVQCQAAGTPLPAPGPVPFLLAPPPPMSAFSPGSSKAAATAPGLAPDAPLDGPMGPTATPGIRPVVQPLQPTSLAQYSTSPFHPLLFFLFTLLTLLNL

>BnLTPG.87

MDPRFCLISALIFSSLLSNSPILILAQISTPCSPTMLSSVTGCMSFLTGGGSSPTSDCCEALKSLTGTGLDCLCLIVTASVPINIPINRTLAISLPRACGMPGVPVKCKASAAPLPAPGPVSLGPTTPPTETQSQGSASFGPTTSPSSSITPDDQNIPASNKGENPTVSTPSGSSPSSSHSLKLPLLLLTFFAFGIINIF

>BnLTPG.88

MEVVRFAVAVVFVLFSVSSSNAEPTPAMGGGAGGGDAHSMPCIQKLMPCQPYLHSVTPPPPASCCLPMKEIVEKDATCLCSVFNNVDMLKSLNLTKENALVLPKACGAKADISLCKSSNGTTTPSTSPGTTMTPPASSTGSGSTGASSSSTAKPTNSAPAIDFAGASFASAFVALATIFF

>BnLTPG.89

MACSSVFITVLISLVPVFLQPGLAQGQSPPASCASLLLALAPCGPFVQGFVQFPAQPCCSSLSQIYSQQPTCLCLFLNNNSILSSAFPINQTLALQLPQLCSVPANSSVCSSGASTASPPSTNSTGSQVSMGAKNNSVAAATPVAQVAPKPSNMMGLGDGLTSSGPKSKIQVTIFVIAAILAGTLFLV

>BnLTPG.90

MKDLHFHIFLVTMTVIASISAATPTAPAAGGALSDECNQDFQKVTLCLDFATGKAPTPSKKCCDAIEDIKEKDPKCLCYVIQQAKTGGQALKDLGVQEVKLIQLPTACQLHNASISNCPNLLGISPSSPDAAVFTSNATTITPEAPAGKSPATPATSSEKGGSASIRDGHAVVALAITLITVSFVSTLPMMALA

>BnLTPG.92

MKMGMGLVFLTVFMAVMSSTRVLAQSTCTSALISMSPCLNYITGNTTSPSQQCCSQLGNVVRSSPDCLCQVLNGGGSQLGINVNQTQALALPRACNVQTPPVSRCNNGGGSTADSPADSPNSSGPGNGSKTVPVGEGEGEGPSSDGSSIKFSYPLLAFLSAASYMAIFLKY

>BnLTPG.93

MEQSTRSLIITIVITSMLAGFGSSDLDQDREECTNQLIVLSPCLTYVGGNAKAPTKDCCGGFGQVITQSQKCVCILVKDKDDPNLGLKFNASLAAHLPTACHITAPNITKCISLLHLSPNSTLAREFESLGRLEASANSAPPSQNVKDGAGGGKAESVKSDGGKKKKSWLAVELLIFALFSHLLLVISSFTSSSFI

>BnLTPG.94

MKQLLVLSNVLLLLSTCDAAAFMSPSESPVSSPSEPSNNDCSTVVYGMFDCLSFLTVGSTDLSPTKTCCEGVKIVLEYNSSCLCVALESSRAMGFDLINNRALAMPSTCNIPIDPHCVSPSKPPTTTPSSGSSPSISTTSPSVPSPACSHSSAAKPGSSPTIIQSPPTLAAPSPAMFAPSPSKSGMENMSVSKLFLIVMMISSFVYLLA

>BnLTPG.95

MAATSNTVVFIVILAITFSSSSETQAPSPPALTCTEELVMFSPCLPYVSAPPNNMSDRPDPLCCSAFSTSAHSGAGNCLCYLLRQPMILGFPLDRSRLLSLSQICSDLSSDESFESICSASESPELPPLQSIQFTAPFVYGMSESISVIVYLNSETRRVSLISAGDTASASPPSLAISREAAGISPTSVQPSPVTDSLSSTPESIINGSPKIRSFWLLSTIIMTLPTSIFTRI

>BnLTPG.96

MIRVMGYNQNRQMLALFITAAIMFLGVRSDLNQDIKGCQDSMSDLYSCLPFVTSKAKAPDSTCCTTLKEKINKGQTKRCLCTLVKDRDDPGLGFKVDANRAMSLPSTCHVPANISQCPEELLHLPPDSVAAKIFKQFTEGLQNVEPRAVPTSSSVKGRDKKQFGLVMAGAFSVWYLV

>BnLTPG.97

MSISIFLGVSTVLAILYAVQATAIWDQDKAMVQCVAKLTPCRPYVNSEAPPPLWCCHPLKKIVENDATCLCEAFKHPDMLALIHLTQEAALNLISSCGASYDASSCNTESSSVSTSKNAALAISLLGVSFVSAFTGL

>BnLTPG.98

MEICKFLTVIFVAVVVLYSVQAAEQGGDHHLMACMQKLMPCQKYIHAVNPPPPASCCGPMKEIVEKDSQCLCTVFNNPALLKSLNLTKENALDLPKACGANPDVSICTKTASSPPIASPTSGGSSVQAVSIIGLAFAFAFVAKILY

>BnLTPG.99

MASSILFVTLLVSLSPIFLQQVLAQVPGTTATCSSMLLSLAPCGPFVQGFVQLPAQPCCDGLNQIYSQQPTCLCLFLNNTSTLSPAFPINQTLALQLPPLCNIPANSSSCTSPGGEAPSDSSSVAPPPSSSTGSQVSPGAKNNSATPVAQLAPRPTSLMGLGYDLRSSGSKSKIQLFILALAPILPGTLLI

>BnLTPG.100

MKGLHFHLFLVTMTVVASVSAATPASPAAGGALADECSQDFQKVTLCLDFATGKAPNPSKKCCDAIEDTKERDPKCLCYVIQQAKTGGQALKDLGVQEDKLIQLPTSCQLHNASISNCPKLLGISPSSPDAAVFTNNATSTTTPVAPAGKSPATPTTSTGTGGSPSIRDGHATVALVLPLMTVSFVSILPRMGLA

>BnLTPG.101

MKKLFVLFIVLLLYSYVAAEFLSPSESPVVSESPLVSPSDAPVVSESPLVSPSDAPVLSESPILSPLGTPVLSPSSEPSNNDCATVIFSMFDCLSFLTVGSKDRSPTKSCCDGVKTVLEYNPNCLCIALESSRDMGFELINRKALAMPSICNIFINPHCDVASSPTASISTPGTTTISPSEPPTNLSPPVVMTPSPPTVTTSSPPAVNAPSPPTFTTPSPPTFTTPSPSVNPSPPAVITSPARTASPPTITHSSQSSQAMTALSPAIIAPSPSKSGATNLSISKLFLRIVTISTFAYVVSFNLI

>BnLTPG.102

MAVTIVIILMLAITFSSSSTAVAETQGPSSSPPAPTCSEELVMFSPCIPYVSAPPNNISKTPDALCCSVFSTSVHSTAGKCLCYLLRQPMILGFPLDRSRLLSLSQICTEFKSSDESFESLCSPSVSPELPPLQSIQFTSPFDYGDRDSASPQSLGLPPETAKISPTSDQFSPDIDNVSPQLIINGSPIISNLLLFLTTIIMTLATSILTRI

>BnLTPG.104

MEIVRFAVIFVLCSISSSNAAITPPSGGAGDAHSMPCIQKLMPCQPYLHSATPPPPASCCMPLKEIVETDVNCLCSVFNNVDMLKSLNLTKENALVLPKACGANADVSQCKASTGTTTPSTSPGTTKTPPASPAESGSTGGPASSTAKPSDSAPAINFSGISFASAFVALATILF

>BnLTPG.106

MEIVRFTVAVFFVLYSVSSSNAATAPPSGGGGGGGDAQAMPCIQKLMPCQPFLHSVIPPPPPSCCLPMKEIVANDATCLCSVFNNVDMLKSLNLTKDNALDLPKACGANPDISLCKASPAGGTTTNSTSPATPKTPPASSTGSGSTGASASSTSTPTSSAPAINFAGLSFASTIVALAATFF

>BnLTPG.107

MIGRFTVSRKTSKKFTVAVFFVLVSVSSSNAAIAPPSGGGSGGGDARAMPCIQKLKSCQPYLHSVIPPLPASCCLSMKEMVANDATCLCSVFNNVDMLKSLNLTKDNALDIPKACGANPDISLCKASPANGTTTNSTSSTPTSSAPAINFAGLSFASTIVALATTFF

>BnLTPG.108

MATATMIFAAAMTAMILVSLQQVEAQPATTCVNKLVPCFSALTTTTKPPKDCCDSIKEAVEDELPCLCTVYNTPGLLSQFNVSTAQALNLSRRCDVTTDLSACSGTGASSPKASLPPPAGNRGNGTGVGNKLAGYGVTTMILSLVSTIFF

>BnLTPG.109

MKQSLLSILILLLSSSFAPIHARNKSQPANSPSLVAAPAPGPSNSDCSSVIYDMMDCLSYITPGSNDTKPTKVCCGGILSVLQYNPTCVCVGLESSKTMGFAVNNTRARAMPTTCKLPIVATHCPMLDEVTPAASSPVSQSAGTPMTSPSSVASPTSSPSLAESPVMTAPSPSSSGTNHLSASTVTLVVIKVSFVAYIAFFFF

>BnLTPG.35

MASSILFITLLISLSSISLQLVFAQVPGTTTATCSSMLLSLAPCGPFVQGFVQLPAQPCCDGLNQIYSQQPTCLCLFLNNTSTLSPAFPINQTLALQLPPLCNVPANASSCSSPGGEAPSDSSSVAPPPSSSTSSPVSPSAKNNSRTPVAQLAPRPTSLMGLGYDLRSSGSKSKIQLIILALAVILPGTLFI

>BnLTPG.78

MEKSTRTLMIITIVITSMLVGFGSSDLDQDREECTDQLIALSTCLPYVGGNAKAPTKDCCGGFDQVITKSEKCVCILVKDKDDPNLGLKFNASLAAHIPTLCHITAPNITKCISLLHLSPNSTLAKEFESLGRIEYEGKTNSTSPSHNVKDGTGGGKAEQVKSTGGKKSWLAVELLIFALFSHLLFIIPSLTSSSFI

>BnLTPG.53

MDPKSFLISALIFSLLSNSPILMSLAQINTPCSPTMLSSATAGCMSFLMGGASSPTSDCCRALKTLTGTSMDCLCLIVTANVPLDLPINRTLAISLPRACGVPVQCKASSALLYAPGPASLGPTTSPPTETQIPEGPASFGPATSPTSSMDPDGMPDDTDVSGPRNGGDPREPPKTSASSPSSSLSLKLSLLLFALFAFEFISFF

>BnLTPG.16

MRSLFLLALFLVLAFHHGEAAVTCNNVVGDLYPCLSYVMQGGNSPSTNCCSGVRTLNSQAQTTADRQSVCRCIKNAIGGASYSSSNLKNALSLPAKCGVNLPFSISPSTNCNSIH

>BnLTPG.30

MKGLQFHLFLVTMTVVVSVSAATPAAPAAGGALADECSQDFQKVTLCLDFATGKAPNPSKKCCDAIEDTKERDPKCLCYVIQQAKTGGQALKDLGVQEDKLIQLPTSCQLHNASISNCPKLLGISPSSPDAAVFTSNATSTTTPAAPAGKSPATPTTSTGTGGSPSIRDGHATVALAFALIMTVSFVSILPRMGLA

>BnLTPG.32

MLKANILTGLLLLFTLSSAQTPPAPEPVAADGPSSPTNCLVSMLNVSDCLSYVQVGSTETKPEAACCPELAGMAQSSPECVCNLLGSGASPRFGVKLDKQRAEELSSICGVKAPSPSLCSVLGFPTISPAGSEDSSSAGSEGSEKDKKNGGMPTKDYGLAFNSLMLALFTIFSLY

>BnLTPG.40

MKKLFVLFIVLLLYSYVAAEFLSPLESPLVSESPLVSPSDAPVVSESPLVLPSDAPVLAESPMLSPLGTPVLSPSSEPSNNDCATVIFSMFDCLSFLTVGSTDRSPTKSCCDGVKTVLEYNPNCLCIALESSRDMGFELINRKALAMPSICNIFINPHCDVASSPTASISTPGTTTISPSEPPTNLSPPVVMAPSPPTVTTSSPPAVNTPLPPTFTTPSPSVNPTPPAVITSPARTASPPTITHSSQSSQAMTALSPAIIAPSPSKSGATNLSISKLFLRIVTISTFAYVVSFNLI

>BnLTPG.42

MVMIYINKVPAAVAIALLFLVVVIAPQWTKAQPLPPFPPMSPLPPLPPMSPLPPLPPMSPLPPLPPMSPLPPMPKVDPVCTTAILDIVQNCYSTLSAVPSEECCNGLKSASKTQVTCLCDNFIAHPVFSNLTRPYYDQVSNACGVLDKYACNGTGEDSKGGDSKGGDPKGGDGNAGAINKIAGSMGLFGLITCLFFLLF

>BnLTPG.82

MESWRISLVAIAIALLMATLVSAGEDKAKDKEECTEQLVGMATCLPYVQGQAKTPTPDCCSGLKQVLKSDKKCLCVIIQDRNDPDLGLQINVSLALALPSVCHAVADVTKCPALLHMDPKSQEAQVFYQLANGLNKSGPGSAPAPTSISPTAGSEDGNNSGRATSLPSKNHAQSFRKRWLVLEVAAHLFIISFTITL

>BnLTPG.105

MEIVRFALAVVFVLYSVSSSNAATAPPSGGGGGDAQAMPCIQKLMPCQPFLHSVIPPPPPSCCLPMKAIVANDATCLCSVFNNVDMLKSLNLTKDNALDLPKACGANPDISLCKASPAGGTTTNSTSPATPKTPPVSSTGSGSTGASPSTTSPTSSAPAINFAGLSFASTIVALATTLF
